# Linking Linguistic and Geographic Distance in Four Semantic Domains: Computational Geo-Analyses of Internal and External Factors in a Dialect Continuum

**DOI:** 10.3389/frai.2021.668035

**Published:** 2021-06-04

**Authors:** John L. A. Huisman, Karlien Franco, Roeland van Hout

**Affiliations:** ^1^ Centre for Language Studies, Radboud University, Nijmegen, Netherlands; ^2^ QLVL, Department of Linguistics, KU Leuven, Leuven, Belgium

**Keywords:** computational sociolinguistics, dialectometry, lexical variation, semantic variation, spatial analysis, mixed-effects regression, limburg

## Abstract

Dialectometry studies patterns of linguistic variation through correlations between geographic and aggregate measures of linguistic distance. However, aggregating smooths out the role of semantic characteristics, which have been shown to affect the distribution of lexical variants across dialects. Furthermore, although dialectologists have always been well-aware of other variables like population size, isolation and socio-demographic features, these characteristics are generally only included in dialectometric analyses afterwards for further interpretation of the results rather than as explanatory variables. This study showcases linear mixed-effects modelling as a method that is able to incorporate both language-external and language-internal factors as explanatory variables of linguistic variation in the Limburgish dialect continuum in Belgium and the Netherlands. Covering four semantic domains that vary in their degree of basic vs. cultural vocabulary and their degree of standardization, the study models linguistic distances using a combination of external (e.g., geographic distance, separation by water, population size) and internal (semantic density, salience) sources of variation. The results show that both external and internal factors contribute to variation, but that the exact role of each individual factor differs across semantic domains. These findings highlight the need to incorporate language-internal factors in studies on variation, as well as a need for more comprehensive analysis tools to help better understand its patterns.

## Introduction

Dialect geography deals with the spatial components of human communicative processes or, on a more abstract level, with the relationship between space and social behavior. Social behavior results in spatial patterns of language variation and change. Languages change as speakers accommodate their speech patterns during interactions with their most common conversational partners—their speech community (Bloomfield, 1933)—and for logistical reasons, these interactions occur more frequently and intensely between people that are geographically close to each other. Previous work has shown that linguistic features first spread across communities that share dense interaction, and then expand into the rest of a language area—a process called diffusion (see Gerritsen and van Hout, 2006, for an overview). As a result of this process, the linguistic varieties of neighboring communities generally differ only slightly (Chambers and Trudgill, 1998). In contrast, contact between geographically distant communities tends to be less frequent. Accommodation therefore occurs to a lesser degree, resulting in communities whose linguistic varieties resemble each other less and less the farther apart they are (Heeringa and Nerbonne, 2001). Linguists often call this gradual pattern a dialect continuum, and many have been studied. For example, Nerbonne (2010) investigated six areas (variation across the Bantu languages, in Bulgaria, Germany, across the United States East Coast, and in the Netherlands and Norway) and found that linguistic distance continuously increases over geographic distance, but that the magnitude of this increase diminishes as geographic distances become larger.

Dialectometry aims to objectively measure linguistic relationships between dialects (Séguy, 1971) and the procedure followed in most studies is based on methods used by the Salzburg school of dialectometry, which compare a geographic distance matrix with a linguistic (dis)similarity matrix (see Goebl, 2006, for an overview). These matrices code pairwise geographic distances and linguistic (dis)similarities between all locations in a language area. The online dialectometry tool *Gabmap* (Nerbonne et al., 2011) developed at the University of Groningen follows this same conceptual structure, separating the geographical and linguistic information in two different matrices. To connect the two, Goebl (2006) refers to the Pearson correlation for comparing linguistic and geographical distances. However, as the assumption of independence of observations is violated when dealing with distance values, Mantel (1967) suggested a permutation technique for evaluating significance in such cases. Nerbonne and Heeringa (2007) used this Mantel test to investigate the correlational structure of the linguistic and geographical distances in the Netherlands. However, subsequent reviews of methods in dialectology appear not to mention the Mantel test again (e.g., Wieling and Nerbonne, 2015), despite its wide application in ecology (for overviews, see e.g., Legendre and Legendre, 2012; Zuur, Ieno and Smith, 2007).

A rather pressing issue with such correlational approaches is how to include external factors other than geography in the analysis. Although dialectologists have always been well-aware of the potential influence of language-external variables such as population size, isolation and socio-demographic features, these characteristics are generally only included in dialectometric analyses afterwards. The interpretative maps produced as output of many Salzburg style studies—and also Gabmap—emphasize the value of visualization. Language-external factors are mostly used for further interpretation of the results rather than as explanatory variables in a more formal, statistical model. In addition, one of the downsides of aggregating measures of linguistic distances is that differences between the linguistic variables involved in computing linguistics distance are smoothed out (e.g., Schneider, 1988). This is especially relevant for measures based on lexical variation, as language-internal semantic characteristics have been shown to affect the distribution of lexical dialect data (cf. Speelman and Geeraerts, 2008; Franco et al., 2019b).

One development put forward to address this is the use of generalized additive mixed-effects regression modeling (GAM) as applied by Wieling (2012), in which both geographic and social predictors are included in a regression design. The analysis is done on linguistic distances between a series of observed points (the dialects) and a reference point (the standard language). Explanatory variables that have been studied using this method include both language-external (e.g., community size, speaker education level), and language-internal factors (e.g., word frequency, grammatical category). The strength of the GAM approach is that the models can include random factors, which allows for more precise control over outlying locations and linguistic items or elements. The GAM has been successfully applied to dialectal variation in e.g., Dutch (Ko et al., 2014), Italian (Wieling et al., 2014), and Catalan (Wieling et al., 2018). Wieling and Nerbonne (2015) provide an overview of other quantitative work on multivariate spatial analysis of language variation, e.g., quantitative counterparts to the traditional identification of isoglosses and dialect regions (Grieve et al., 2011).

The GAM approach clearly shows the advantage of regression analysis in explaining linguistic variation, but one particular limitation is the use of a single reference point in defining (dis)similarity. While the analysis provides valuable insights into which factors play a role in differentiation from the standard (the reference point), it is more limited in exposing overall patterns of variation that exist in the dialect area as a whole, i.e., how variation between non-standard varieties is patterned. In addition, when dialect areas are spread across multiple countries—such as Limburgish, the area under investigation here—it is even harder to determine what to use as a reference point. In dialectometry however, we prefer to compare each location to all other locations. This gets rid of the need for a single point of reference, and it helps understand the patterns of linguistic variation across the entire linguistic area. This challenge was recognized by Wieling and Nerbonne (2015), who advocated the search for an approach that is able to incorporate information from the linguistic landscape as a whole, while at the same time including non-linguistic factors as explanatory variables. In this paper, we showcase two regression methods that keep all location-by-location comparisons intact.

As a first step, we use Multiple Regression on distance Matrices (MRM; Lichstein, 2007). MRM is an extension of the (Partial) Mantel test on two (or more) distance matrices. In essence, the relationship between the Mantel test, the partial Mantel test, and MRM is the same as between analyses of correlations, partial correlations and multiple regression. The main advantage of MRM over the Mantel test is that while the Partial Mantel test combines the different explanatory factors into a single distance matrix, MRM allows for each factor to be included individually, which makes it possible to assess their individual importance. As such, it is more flexible in terms of the types of data that may be analyzed (e.g., binary, continuous), and it provides estimations of explained variance. In addition, significance testing is done on the basis of random permutations to avoid overestimating the significance of the correlations. In contrast to earlier studies using GAM with a single point of reference (e.g., Wieling, 2012; Wieling et al., 2014; Wieling et al., 2018), the use of distance matrices allows to include the distances between all locations in a single analysis.

We use the MRM analysis as an in-between step toward a more comprehensive analysis based on linear mixed-effects modeling (LMER). LMER shares with GAM the potential to include random variables, and was applied by Huisman, Majid and van Hout (2019) to analyze the role of external factors in linguistic variation across Japan. Here, we expand on this by applying the method to a much larger dialect database for the Limburgish dialect continuum in the Netherlands and Belgium, and by including language-internal factors. Crucially, LMER renders the use of the location-by-location matrix format obsolete, which is particularly important for variables that cannot be coded into distance matrices, i.e., language-internal factors such as semantic density and salience. We performed a simulation study to show the strength of linear mixed-effects models (LMER) in comparison to Multiple Regression on distance Matrices (MRM), which we describe in the Supplementary Material.

The main aim of this contribution is to chart the many promising possibilities of our application of linear mixed-effects regression modelling (LMER) on full pairwise distance matrices to simultaneously investigate external and internal drivers of language variation. By incorporating techniques from quantitative ecology into the dialectometric methodology, we can develop strong explanatory models of patterns of language variation on a large spatial scale. Following dialect geography, which deals with the intertwining of linguistic and geographical variation, the primary link in explaining differences in a dialect continuum is geographic distance, an external factor. However, we will show that geographic distance is the entrance to test the role of additional factors, internal or external. Our implementation of LMER provides a more comprehensive analysis tool that offers supplementary techniques to analyze and understand patterns of geographical language variation, that seem to be superior in several respects to techniques currently used in dialectometry.

## Materials and Methods

### The Limburgish Dialect Continuum

**FIGURE 1:**
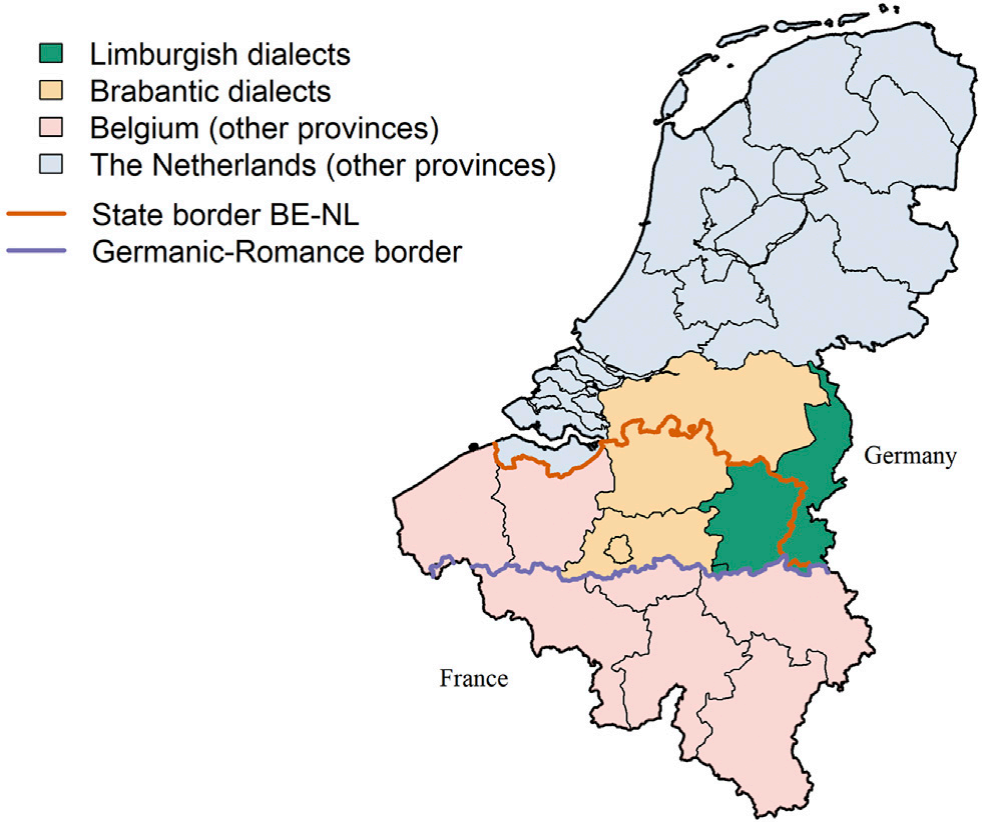
The Limburgish dialect region (in green). The brown line within the green area is the boundary between the Dutch (on the **right**) and the Belgian (on the **left**) province of Limburg. The purple line in the south of the dialect region shows the boundary between the Dutch-speaking (northern) and French-speaking (southern) part of Belgium. The other provinces of the Netherlands and Belgium are colored light-blue and pink.

**FIGURE 2:**
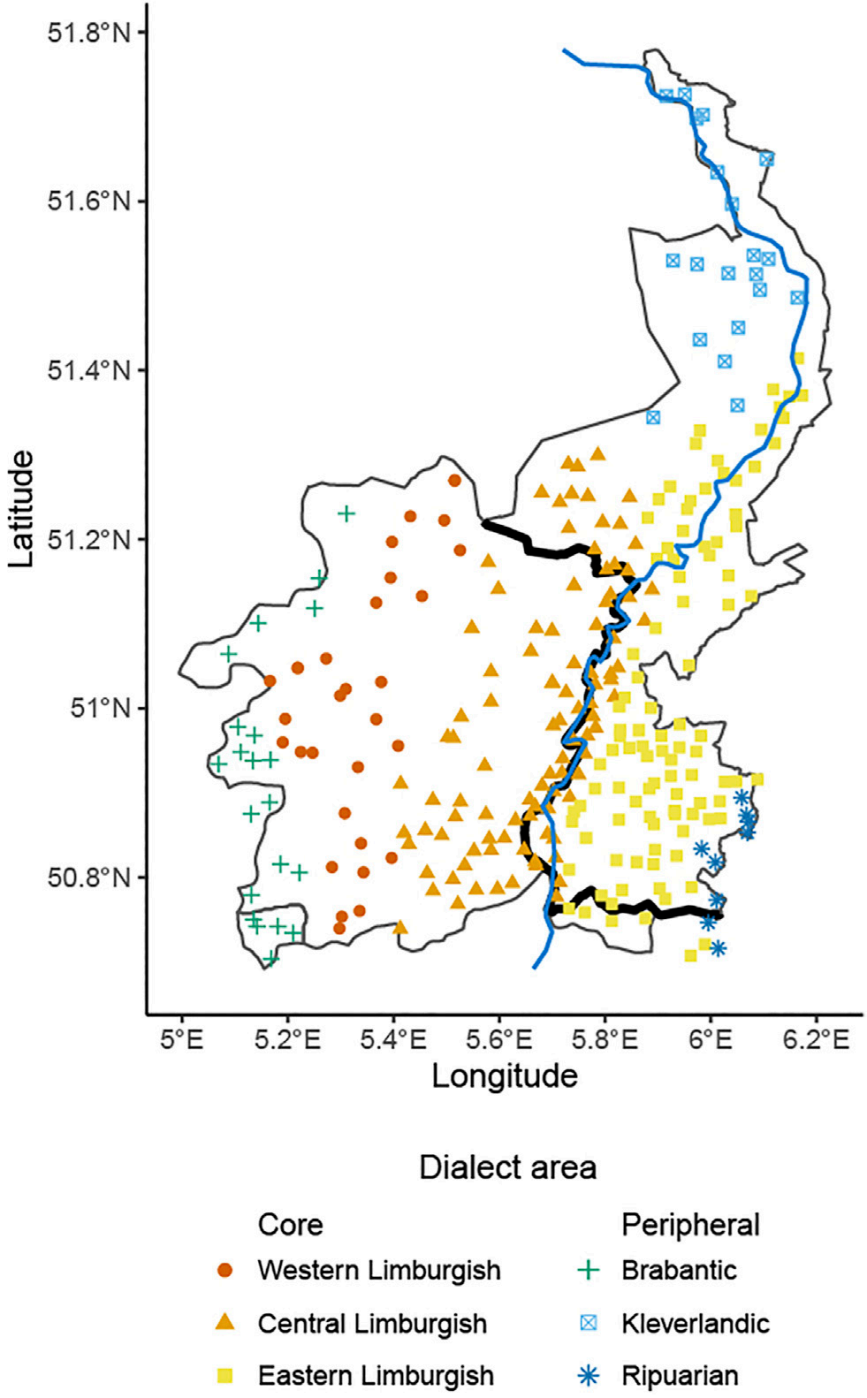
Map of locations included in the database, with their classification into one of six dialect areas.

The lexical data analyzed in this paper comes from the Limburgish dialects, spoken in the northeast of Belgium and in the southeast of the Netherlands (marked in green on Figure 1). In the south, the dialect area is demarcated from the Romance language area by the Germanic-Romance border (marked in purple). In the east, the dialect area is demarcated by the national border with Germany. The German and Dutch dialects (which include the Limburgish dialects) historically form a dialect continuum—some of the dialects spoken in the south of Limburg (e.g., the Ripuarian dialects, see Figure 2) in the Netherlands can even be considered dialects of German as they underwent the second Germanic consonant shift. In the north and west, the dialect area borders the Brabantic dialect area, another dialect area of Dutch (marked in orange on Figure 1). Although there is some discussion about where the Limburgish dialects end and the Brabantic dialects begin, the demarcation is often equated with the provincial borders in the Netherlands and in Belgium (Weijnen, Goossens and Goossens, 1983). Thus, it is accepted that Limburgish dialects are spoken in the Belgian province of Limburg and in the province of Limburg in the Netherlands, whereas Brabantic dialects are used in the Belgian provinces of Flemish Brabant (including Brussels) and Antwerp, and in the province of North Brabant in the Netherlands.

Six subregions can be distinguished within the Limburgish dialect area: Western Limburgish, Central Limburgish, Eastern Limburgish, Kleverlands, Ripuarian, and Brabantic (Van de Wijngaard and Keulen, 2007). The latter three areas are peripheral and transitional areas that share a border with other dialect regions. In addition, the national border between Belgium and the Netherlands runs through the dialect area. Its current position was officially determined in 1839 when the independence of the nation of Belgium was definitively recognized internationally.

### The Dictionary of the Limburgish Dialects

The linguistic data we used come from the *Dictionary of the Limburgish Dialects*. These data offer a firm basis to gain insight into patterns and processes of linguistic variation and change. They cover a large part of the lexicon, comprising thousands of concepts belonging to all aspects of human life. Importantly, the data were collected at the concept level, avoiding possible bias in the data selection process as the researcher does not need to determine which variants are synonymous across locations. The data were collected highly systematically, mainly through large-scale dialect questionnaires distributed between 1960 and 1990, in which lexical variants were elicited for every concept. In addition, the paper version of the dictionary contains data from additional sources (e.g., local dialect dictionaries and specialized terminological dialect collections), which was used to complement the dictionary entries.

For our analyses, however, we only used the data that were collected by means of the questionnaires. Furthermore, we used the digitized version of the dictionary, which has in recent years also become available online (http://www.e-wld.nl), as the paper dictionary does not contain all the questionnaire data as a result of editorial work. For example, concepts without any variation across the entire dialect area, or questions that produced messy data as respondents found them difficult, were not included in the paper dictionary. However, for our analysis, we use all the data available in the database, including concepts without variation or with messy responses.

### The Four Semantic Domains

The dictionary is divided into three large parts, covering all aspects of human life in the first half of the 20^th^ century: agrarian terminology, non-agrarian professional terminology, and general vocabulary. Every part consists of about a dozen volumes, each containing the vocabulary for one specific semantic domain. In the analyses presented here, we focus on four semantic domains from the general vocabulary: *Church and religion*, *Clothing and personal hygiene*, *the Human body*, and *Society and education*. These domains were selected because the concepts they represent vary along two axes. First, they differ in the degree to which they contain basic vocabulary concepts (*the Human body*) versus culturally variable concepts (*Church and religion*, *Clothing and personal hygiene*, and *Society and education*). Second, the semantic domains with cultural vocabulary show differing degrees of top-down standardization. For example, the *Church and religion* field is characterized by a high degree of standardized vocabulary, often of Latin origin, related to general religious practices and traditions. In contrast, there is no high degree of standardization for the *Clothing and personal hygiene* field. In addition, the *Church and religion* domain contains concepts relating to the Catholic church in particular, but as will be explained below, the Catholic religion does not play an equally large role throughout the dialect area.

**FIGURE 3:**
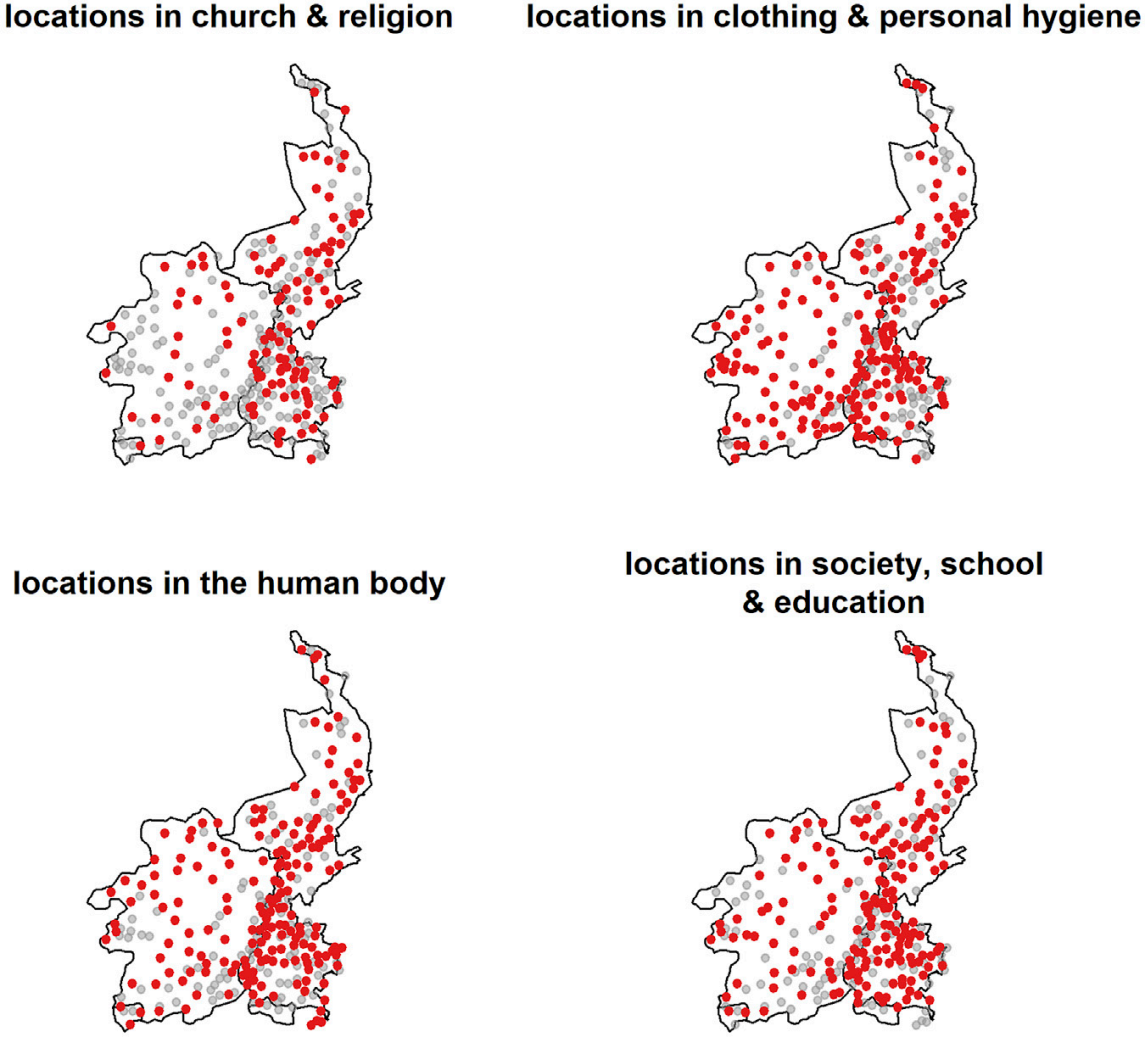
Locations available per semantic domain (in red). Grey circles indicate that no data is available for a particular semantic field.

Table 1 provides an overview of the data per domain in the database. In most semantic domains, data is available for 175 or more locations in the Limburgish dialect area, but this number is lower in *Church and religion* (114), where the distribution of locations with data is less dense across the dialect area (see Figure 3, where locations without data per semantic field are shown with a grey circle, whereas locations with data are marked in red). In addition, Table 1 also presents the information of the language-internal factors that will be included in the analysis below. The domains and these language-internal factors are discussed in more detail in the following sections.

**TABLE 1:** Data per semantic domain in the database.

Semantic domain	Number of locations	Number of concepts	Number of subsections per level of depth			Ratio of multi-word concepts	Concept length	
			1	2	Max		Mean	Median
Church and religion	114	592	5	19	35	0.30	12.9	11
Clothing and personal hygiene	188	323	5	18	43	0.48	13.8	12
Human body	179	180	3	14	18	0.32	10.7	9
Society and education	175	462	4	19	55	0.21	9.9	9

#### The *Church and Religion* Domain

The *Church and religion* domain contains concepts relating to different aspects of Catholicism. It consists of five subsections: *In and around the church building*, *Liturgy and devotion*, *Catholic Holy Days and rites*, *Catholic belief and faith*, and *The clergy*. Table 2 contains example concepts for each subsection. The first subsection, *In and around the church building*, consists of concepts relating to the interior and exterior of a typical church building in the Low Countries—e.g., the names for the typical parts of a church, such as the baptistery, the sacristy, the church tower and bells and the cemetery that is typically found around a Catholic church. The second subsection, *Liturgy and devotion*, mostly contains concepts relating to the Catholic mass, such as its different types (e.g., in the morning, at night, for children), its typical parts, and different prayers (e.g., the Lord’s Prayer, Hail Mary). The third subsection, *Catholic Holy Days and rites*, contains concepts relating to the Catholic Holy Days and the Catholic Calendar (e.g., Christmas, Easter), and also describes the seven sacraments (i.e., baptism, marriage, confession, etc.), as well as the Catholic funeral. The fourth subsection, *Catholic belief and faith*, contains more general aspects of Catholic faith, such as religious concepts (e.g., purgatory, fallen angel, miracle), as well as different virtues and sins. The final subsection, *The clergy*, contains the different names for people belonging to the clergy.

**TABLE 2:** Examples of concepts in the subsections of the *Church and religion* domain.

Subsection	Examples
In and around the church building	Church, leaded window, credence (table), church bell, tombstone
Liturgy and devotion	Early mass, offertory, holy water, rosary, to pray
Catholic holy days and rites	Patron saint, advent, good friday, confirmation, confession
Catholic belief and faith	Catechism, devil, baby jesus, fasting
The clergy	Pope, franciscan, monk, dean

Previous research on this semantic domain in the *Dictionary of the Limburgish Dialects* and its counterpart for the Brabantic dialects has shown that there is a large number of loanwords from Latin, traditionally an important language of the Catholic church (Franco et al., 2019a). The frequency distribution of Latin loanwords across the two dialect areas was largely similar, with only minor differences between Brabant and Limburg. This indicates that these loanwords were not distributed across the dialect areas by linguistic diffusion, as this would have resulted in a more wave-like pattern of their distribution. Instead, the loanwords were likely introduced in all dialects as necessary borrowings for religious concepts and then transmitted from generation to generation (cf. Labov, 2007). These findings are not surprising as the Catholic religion has held a strong position in the Low Countries—especially in the south of the language area, which includes the Limburgish and Brabantic dialects. For example, data from Schmeets (2014:6) indicates that at the beginning of the 20th century (in 1909), 100% of the people living the Dutch province of Limburg self-reported as being Catholic. In1987,^1^ close to the time when most of the data for this semantic domain was collected, this number had only dropped to 89%. Due to the fact that the rites and structure of the Catholic church are standardized, we may also expect that the effect of geographic distance in this semantic domain is smaller than in other semantic domains.

#### The *Clothing and Personal Hygiene* Domain

The *Clothing and personal hygiene* domain consists of five subsections: *Clothing*, *Headgear*, *Foot- and legwear*, *Jewelry and ornaments*, and *Personal hygiene*. Example concepts for each subsection are shown in Table 3. Most concepts belong to the first subsection, *Clothing*. The semantic domain as a whole contains culturally variable vocabulary, as evidenced, for instance, by the fact that some of the concepts have fallen out of use more recently (e.g., jerkin, nightcap). Concepts related to clothing have been shown to be prone to lexical borrowing (Tadmor, 2009) and previous work has confirmed that this is also the case in the Limburgish data. Many loanwords from French are in use, especially in the Belgian part of the dialect area (Franco et al., 2019a). We may therefore expect that this domain is prone to patterns of diffusion (Labov, 2007), resulting in larger linguistic differences between locations that are further away from each other.

**TABLE 3:** Examples of concepts in the subsections of *Clothing and personal hygiene* domain.

Subsection	Examples
Clothing	Smock, undervest, to fit, women’s coat
Headgear	Beret, hat, pom-pom of a bonnet, bowler hat
Foot- and legwear	Barefoot, women’s shoe with medium or high heel, clog, sock
Jewelry and ornaments	Watch, medallion, jewelry, sequin
Personal hygiene	To shower, to brush teeth, toothpick, razor

#### The *Human body* Domain

The *Human body* domain consists of three main parts: *The body and its parts*, *Organs and their functions*, and *The senses*. Example concepts for each of these parts are provided in Table 4. Many body part concepts have been included in basic vocabulary lists. For instance, 19 concepts on the 100-item Swadesh list (Swadesh 1955) and 25 concepts on the 100-item Leipzig-Jakarta list (Tadmor, 2009) are part of this domain. However, the dictionary also contains entries for several jocular terms for body parts (e.g., for the head or mouth), children’s names for body parts, taboo meanings (e.g., names for male and female genitalia), as well as concepts that are cognitively less salient, referring to parts of the body that might not turn up often in everyday conversations (e.g., dimples, or the upper part of the back). For the basic vocabulary concepts, we expect to find little lexical variation, whereas more variation can be expected across the dialect area for the jocular terms, children’s names, and taboo and non-salient concepts (see Speelman and Geeraerts, 2008; Geeraerts and Speelman, 2010; Franco et al., 2019b).

**TABLE 4:** Examples of concepts in the subsections of the *Human body* domain.

Subsection	Examples
The body and the body parts	Short, curly hair, eye, navel
Organs and their functions	To breathe, stomach, kidney, diaphragm
The senses	To see, to wink, flavor, to listen attentively

#### The *Society and Education* Domain

The *Society and education* domain consists of four diverse subsections relating to the different aspects of public life: *People and society*, *Societal organization*, *Transport*, and *School and education*. Table 5 shows examples for each subsection. The first subsection, *People and society*, is the largest and comprises topics on social life in the community (e.g., names for neighbors and visitors, going to parties, and friendship and animosity), trade (e.g., buying and selling, names for monetary units, names for commercial places, names for property), social etiquette, as well as language and communication. The second subsection, *Societal organization*, describes topics such as the organization of the state, policing, the judiciary, and war. The third and fourth subsections, *Transport* and *School and education*, are more limited in size, containing concepts relating to different modes of transportation (e.g., by air, water or road), and the organization of the education system. As that many concepts in this broad domain are related to the way a state is organized, we may expect that state-level decisions (e.g., the monetary unit that is used in a particular country) lead to small differences between locations even when they are geographically far away. However, larger differences between locations may be found for concepts that describe aspects of societal organization at a lower level (e.g., neighbors, staying out late). Finally, as the Limburgish dialect area spans two countries, the national border might play a large role in variation for this domain.

**TABLE 5:** Examples of concepts in the subsections of the *Society and education* domain.

Subsection	Examples
Man and society	Company, to peddle, night owl, market booth, (Dutch) guilder, impolite, fairy tale, to complain
Societal organization	Mayor, liberal, charge, perjury, soldier, to fall in battle
Transportation	Pedestrian, women’s bicycle, train, steamboat, airplane, to travel
School and education	Boarding school, teacher, ruler, report card

### Explanatory Factors

In the analyses, we examine the effect of both internal and external factors on linguistic distance between locations. We selected five language-external and two language-internal factors that have been previously used in dialectology and related fields to predict linguistic variation and change, each of which is described in detail below.

#### Language-External Factors

##### Geographic Distance

The first factor we investigated is geographic distance. This factor has a well-known effect on linguistic distances: the further two locations are located from each other, the more their language is expected to be different (e.g., Bloomfield, 1933; Chambers and Trudgill, 1998). Geographically, this results in linguistic structures being distributed across a dialect area in a wave-like pattern. An explanation for this finding is the frequency of contact between language users—the principle of density of communication (see Bloomfield, 1933).

For the analyses, we determined latitude and longitude coordinates for all locations in our dataset and calculated straight line geographic distance in kilometers between every pair of locations. As logarithmic distance has been shown to predict linguistic differences more accurately in several studies (e.g., Nerbonne and Heeringa, 2001), we also calculated the natural logarithm of the geographic distances.

##### Separation by Water

Another factor that has been argued to affect linguistic distance is isolation: the more isolated a speech community is (e.g., due to natural borders such as rivers or mountain ranges), the less similar their language will be to other related surrounding varieties. Water as a natural border has been shown to influence variation in the Dutch language area. For instance, the word for “purse” differs across the islands of the Dutch province of Zeeland: *borre* is used on the island of Goeree, *bozze* on Schouwen and Southern Beveland and *beuze* on Walcheren and Noord-Beverland (Weijnen, 1966). This effect of separation by water has also been shown across the Japanese archipelago, for both varieties of Japonic (Lee and Hasegawa, 2014; Huisman et al., 2019) and Ainu (Lee and Hasegawa, 2014).

Through the Limburgish dialect area runs the river Meuse, which partly forms the border between Belgium and the Netherlands. In contrast to oceanic barriers discussed above, the role of rivers in creating isoglosses in dialect areas is less clear. A river can both separate and unite depending on its navigability (Weijnen, 1966), which is why it is interesting to include the Meuse in the current study. For the analyses, we first determined, for each location, whether it is located to the west or to the east of the river and then coded, for each pair of locations, whether they are located on the same side of the Meuse (coded as 0) or on opposite sides (coded as 1).

##### Population Size

Another factor that has been shown to influence linguistic distances is population size. In Trudgill’s gravity model, for example, the effect of geographic distance is mediated by population size: language changes are expected to first diffuse from one large city to another, and to only be adopted later in smaller locations (Trudgill, 1974; Chambers and Trudgill, 1998). The explanation is again contact between speakers: the language used in large urban centers influences the language of smaller locations.

Because the locations available in the *Dictionary of the Limburgish Dialects* are often neighborhoods or districts that are part of a larger administrative community, obtaining accurate and specific population sizes is not without challenge. Especially in Belgium, figures are only publicly available at the administrative community-level. In addition, the questionnaires for the dictionary were distributed several decades ago and obtaining public *historical* population data for every location in the database is even more difficult. Finally, many administrative communities were merged in Belgium in the last quarter of the 20th century (i.e., after the dictionary project started; see De Ceuninck, 2009). For example, Heusden and Zolder in the center of Belgian Limburg were historically two separate administrative communities but merged into a single administrative community (Heusden-Zolder) in 1977.

To handle these challenges, we opted for a systematic procedure that ensures that the population size data we collect is as comparable as possible across the two countries and the dialect area as a whole.^2^ For most of the data, we can rely on census data collected by the national governments. The oldest data available in Belgium stems from 2008 (Stat Bel, 2021), and so we also used data from 2008 in the Netherlands (CBS, 2021). While the Dutch data also provides population sizes for districts and neighborhoods *within* an administrative community (CBS, 2017), we have not found the same type of information for the locations in Belgium. For locations for which data was not directly available, we used information from Wikipedia.^3^ Finally, for 8 locations (1.2% of the total) we did not find any figures for their population size. The median population size of all locations is equal to 2,242. Since the locations without population sizes are generally small, we rounded this number down to 2,000.

As the techniques we used require distance matrices as their input (see *Statistical Analysis*), we calculated the (absolute) *difference* in population size between all pairs of locations in the database. In addition, to account for magnitude effects, we performed log transformation on these differences. We decided to include population size as an individual predictor rather than a gravity-based approach because this has the potential to determine the strength of population size on its own—which is important given that previous work has shown that in some cases the bulk of the influence exerted by gravity comes from distance alone, e.g., Nerbonne and Heeringa (2007).

##### National Border

The third factor we investigated is the national border between Belgium and the Netherlands. The effect of national borders is well-known in the dialectological literature and may lead to dialect divergence due to convergence with the national language (Hinskens et al., 2000). Dutch is a pluricentric language, with Netherlandic and Flemish standard varieties (Willemyns, 2013). It has been shown that the border affects the dialect variants used (Cajot, 1977; Gerritsen, 1999; Franco et al., 2019a).

For the analyses, we first determined, for each location, whether it is located in Belgium or the Netherlands, and then coded, for each pair of locations, whether they are located in the same country (coded as 0), or in different countries (coded as 1).

##### Dialect Area

Finally, to control for potential effects of increased uniformity within dialect areas (see e.g., Shackleton, 2005; Nerbonne, 2013 for previous uses of dialect area as explanatory factor), we classified each location into one of six subgroups based on the classification from Van den Wijngaard and Keulen (2007), as outlined above: the three core areas Western Limburgish, Central Limburgish, and Eastern Limburgish, and the three peripheral areas Brabantic, Kleverlandic, and Ripuarian—see Supplementary Table S3 for the classification of each location. For the analyses, we then coded, for each pair of locations, whether they are from the same dialect area (coded as 0), or from different areas (coded as 1).

#### Language-Internal Factors

##### Semantic Density

Semantic density concerns the extent to which a semantic domain is carved up into lexicalized concepts by language users. Some semantic domains are very dense with many different meanings being lexicalized, whereas other domains use semantically broader and vaguer concepts. Semantic density can differ across languages. For instance, Majid and Burenhult (2014) showed that very few olfactory concepts are lexicalized by speakers of English, but that speakers of Jahai, nomadic hunter-gatherers of the Malay Peninsula, can name and distinguish smell as easily as color, as smell takes up a prominent place in their everyday life and communication. While this shows that, at least at the broader culture level, semantic domains can differ in density due to differences in communicative needs (e.g., Kemp et al., 2018), little research exists on such differences between domains within a dialect area. We therefore included this variable in the analyses to test whether semantic density correlates with linguistic distance. Following the results of previous work, we presume that increased cultural relevance leads to increased semantic density, which in turn leads to decreased lexical variation.

For the analyses, we used two approaches to represent semantic density (see Table 1). First, we determined, for each domain, the number of subdomains into which each respective semantic domain is divided, by using the subsections identified in the dictionary. We selected three levels of granularity: the first (broadest) level of subsections, the second level of subsections, and the deepest level (ranging between 4 and 6, depending on the domain). Secondly, we determined the total number of concepts in each domain, and calculated the average number of concepts per subsection at each level of granularity.

##### Salience

Salience concerns the extent to which a particular meaning is familiar to language users. For example, concepts like pants, shoe or shirt are highly salient: most human beings in industrialized societies are probably familiar with them and come into contact with them every day of their life. In contrast, concepts like jerkin or bowler hat are much less salient (at least nowadays) because these concepts represent objects that people no longer make use of often.

The notion of salience was introduced in Geeraerts et al. (1994), who relate it to the basic-level hypothesis (Berlin, 1972, Berlin, 1978; Berlin et al., 1973). This hypothesis is based on the fact that, cross-linguistically, folk biological classifications consist of a limited set of taxonomical levels, which reflect the degree of salience of the organisms involved. Referents with a high degree of salience (e.g., oak, robin), constitute the core of any folk biological organization and, thus, the basic level: “[a]t this rank, both plants and animals appear perceptually most distinct to the human classifier, and these differences in morphology and behavior virtually ‘cry out to be named’” (Berlin, 1978: 24). Properties of categories at the basic level are that their name is highly frequent (Rosch et al., 1976) and typically consists of a short, primary lexeme, i.e., a non-compositional simplex word like *oak* or *robin* (Berlin, 1972: 54).

Geeraerts et al. (1994) showed that the concept of the basic level is problematic when applied to other types of categories, like artefacts such as clothing. First, the hypothesis presupposes a neat taxonomical organization of the lexicon, because it is based on inclusion relationships. However, a clothing item like *broekrok* “culottes, lit. pants-skirt” poses a problem in this view as it is difficult to place in a taxonomy in which skirts and pants form the basic level. The authors argue that the lexicon is organized in the form of overlapping taxonomies that are all based on different dimensions. Secondly, and more importantly, Geeraerts and colleagues show that for artefacts like clothing items, onomasiological typicality exists between categories *on the same level* of a taxonomical hierarchy as well. For this reason, they propose to take into account a generalized notion of onomasiological salience, which they relate to Langacker (1987) notion of entrenchment. Crucially, this approach allows them to show that differences in salience, both between and within taxonomical levels, correlate with naming preferences, including the fact that concepts that are more entrenched are more likely to be named with simplex forms. Later studies have shown that concepts with a higher degree of salience not only correlate with naming preferences, but also with decreased dialectal variation (Speelman and Geeraerts, 2008; Geeraerts and Speelman, 2010; Franco et al., 2019b). For example, in the semantic field of the human body, the Limburgish dialect dictionary only contains a single word for a highly salient concept like blood, whereas a lot more variation occurs for less salient concepts like the little dents between the knuckles, or bristly (w.r.t. hair on one’s head).

For the analyses, we used two aggregate measures to determine the average degree of salience per domain, which we based on the concept names available in the dataset (see Table 1). These were usually the prototypical Standard Dutch word for that concept. First, following the tendency for high salience concepts to be named with non-compositional simplex words, we calculated, per domain, the ratio of multi-word concepts compared to the total number of concepts. Secondly, following the correlation between salience and word length that has been described, we calculated the mean and median concept length in number of characters [a similar approach was used in Franco et al. (2019b), Speelman and Geeraerts (2008), and Geeraerts and Speelman (2010)]. We expect that linguistic distances will be larger in semantic domains with more multi-word concepts or with longer concepts.

### Statistical Analysis

#### Linguistic Distances

Several measures of linguistic distance have been used in dialectometry, such as the binary same vs. different coding, or Levenshtein distance, to calculate how much two forms differ from each other (see Nerbonne and Kleiweg, 2007 for a discussion). The data we extracted from the *Dictionary of the Limburgish Dialects* uses a standardized coding system based on cognacy, which the editors of the dictionary invested based on their expertise in dialectology and historical linguistics (van Hout et al., 2014). A major advantage of coding entries on the lexical level was that it was no longer necessary to make (sometimes rather arbitrary) decisions about the level of phonetic detail to be coded. This is especially relevant because in this case, the volunteers filling in the many dialect questionnaires were not linguistic professionals. As a result, specific surface forms are collapsed into a general entry. For example, *toŋ* (Maastricht dialect), *tuŋ* (Hasselt dialect) and *tsoŋ* (Kerkrade dialect) for “tongue” are all in the dictionary as tong. This has consequences for measuring distances. The finer phonetic details are not transcribed in the standardized forms. This is likely why preliminary results showed that, based on measures of explained variance and residual scores, string edit distance algorithms were outperformed in our data by methods for binary distance coding.

As the quality of these dictionaries is thus found at the *lexical* level, we used a measure based on the Weighted Identity Value (*Gewichteter Identitätswert*, GIW; Goebl, 1984), which codes binary differences, but takes into account how frequent particular word forms are and weighs them accordingly. We used Gabmap (Nerbonne et al., 2011) to calculate linguistic distances between all pairs of locations based on Gabmap’s use of *d = 1-GIW*—a measure we will call Weighted Dissimilarity Value for the remainder of this paper—for each semantic domain separately.

For the regression analyses, we also computed the logit value of the distance measure to tackle two problems: 1) the range of the dependent variable, 2) the non-linear relation between this variable and the natural logarithm of geographic distance.

#### Correlational Analyses

To assess how patterns of linguistic variation can be explained by language-external factors, we used Mantel correlations, which are widely used in ecology to analyze relations between measures coded in pairwise distance matrices. The analyses were performed in *R*, using the *ecodist* package (Goslee and Urban, 2007). We used the *mantel* function to calculate partial Mantel correlations (using 10,000 permutations and 1,000 bootstrap iterations on 95% confidence intervals) between linguistic distances and each of the language-external factors—for each semantic domain separately. In addition, we used the *MRM* function to perform Multiple Regression over Distance Matrices (using 10,000 permutations) for each domain separately.

#### Regression Analyses

The need for data coded as distances matrices required for analyses based on Mantel correlations brings about several shortcomings. One factor that cannot be addressed using such correlation analyses is the inherent uniqueness that individual varieties included in this (or any linguistic) study all possess. We believe that taking this individual variability of dialects into account allows for better estimation of the contribution of explanatory variables (both external and internal), which is why we used linear mixed-effect regression (LMER) modelling to repeat the MRM analyses, adding to this each individual location as a random factor to account for their inherent uniqueness.^4^

In addition, the language-internal factors we are examining in the current study (see above) do not contain information specifically about individual dialects. This means they cannot be coded into distance matrices either and require the use of other methods as well. Again, linear-mixed effects modelling can incorporate such variables, and as such we performed a series of models with the language-internal factors included to assess their contribution to patterns of linguistic variation.

We performed the analyses in R, using the *lme4* package for the modelling, the *reghelper* package to calculate standardized coefficients, the *lmerTest* package for estimates of *p*-values, and the *piecewiseSEM* package to derive pseudo-R^2^ values.

## Results

### Overview

**FIGURE 4:**
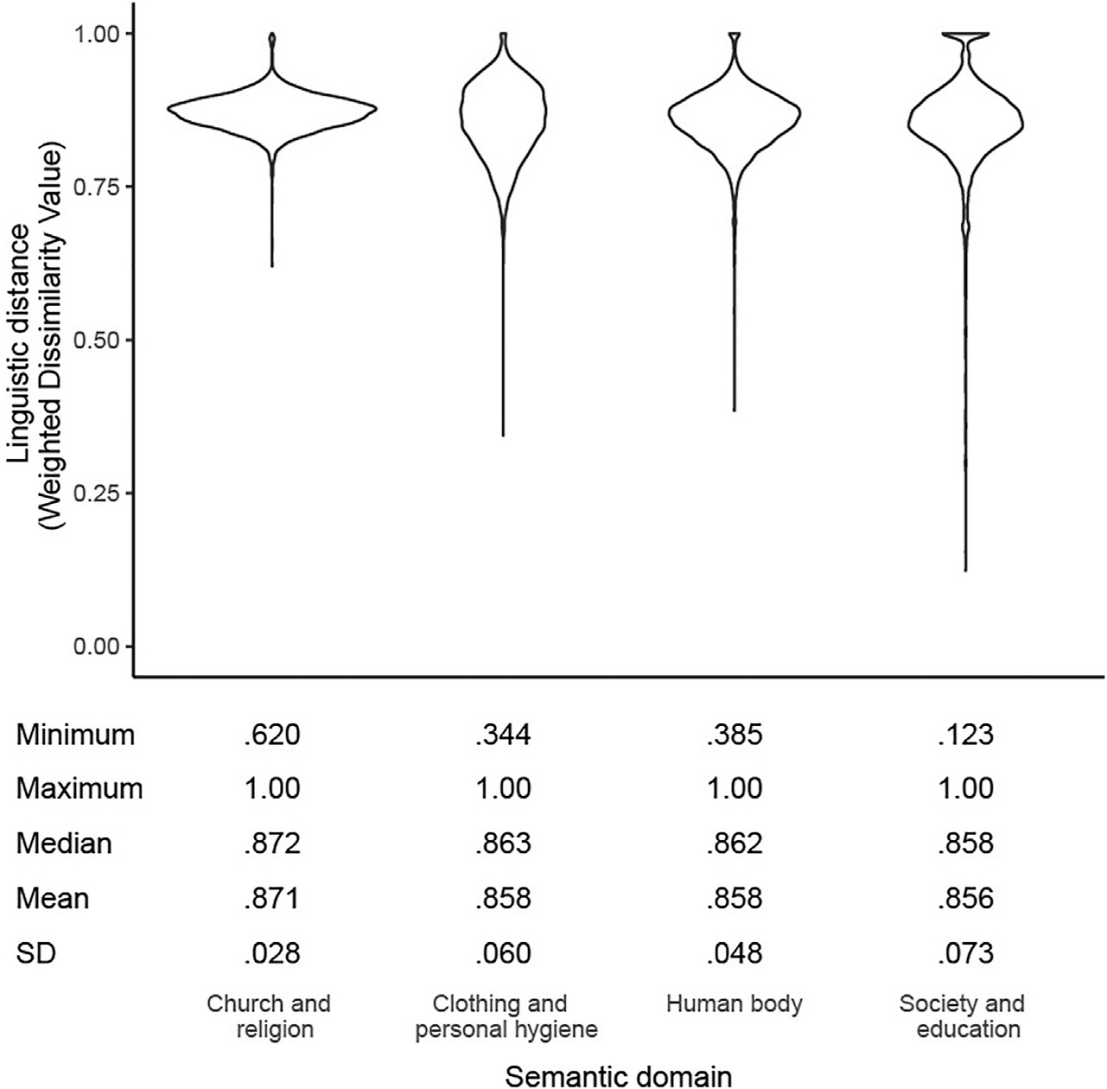
Distribution of linguistic distances across four semantic domains.

Figure 4 is a violin plot of linguistic distances (as measured through the Weighted Dissimilarity Value) for all unique pairwise location-by-location comparisons across the four domains—excluding comparisons with the same location. The figure shows that even though the mean Weighted Dissimilarity Value was virtually the same across the four domains (0.86–0.87), the range and distribution of linguistic distances differs considerably between them, indicating the value of conducting analyses on a domain basis. The scores are fairly high, indicating a high degree of lexical variability, which may in part be due to the many multi-word answers to the questions in the dialect questionnaires. These multi-word responses can come about in several ways. Sometimes the question that is asked to elicit dialectal responses will by nature elicit a multi-word form. For example, for “a note of 100 franc” multi-word responses, such as *biljet van honderd, briefje van honderd* or *bankje van honderd frank*, are elicited*.* Similarly, the question “to change your mind” occurs many times with the reflexive pronoun *zich* (specifically in the construction *zich bedenken*). In these cases, the linguistic distances between the words will be small, as identical multi-word responses are also aligned through the Gabmap algorithm. However, in other cases, these multi-word expressions are a sign that the language user is not familiar with the dialect word for the concept in their dialect, and uses a more descriptive response. For instance, for “to grin,” one respondent from Susteren used the description *uitgestreken gezicht* (lit. “a straight face”). If many respondents use a large set of these types of descriptive multi-word responses, the linguistic distances will be very large. The effect of these types of multi-word responses on dialect variability is discussed further in Franco et al. (2019b).

**FIGURE 5:**
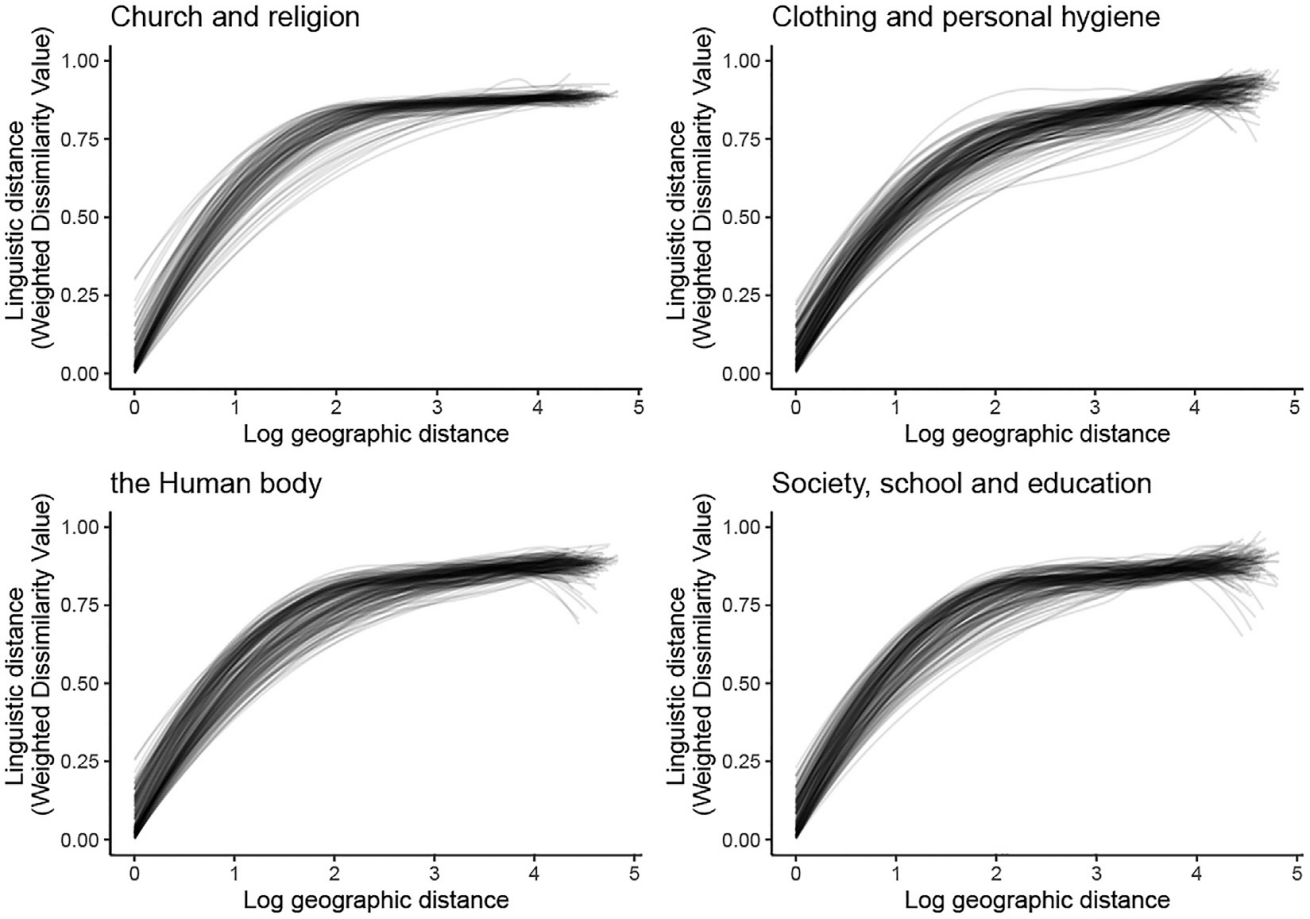
Linguistic distance over geographic distance across four semantic domains.

Figure 5 illustrates the overall relationship between geographic distance and linguistic distance across the four domains. We plotted, for each location, a LOESS smooth of linguistic distances as a function of geographic distance and included the smooths for all locations into a single plot per semantic domain, including the comparison of a location with itself. As the figure shows, the overall trend is similar across the four domains, but there are some differences between the individual smooths, indicating that the relationship between geographic distance and linguistic distance differs across both locations and domains. For example, the curves show that lexical dissimilarity rapidly increases over distance in the beginning, but this increase is more pronounced for the *Church and religion* than for the *Society and education* domain. In addition, where linguistic differences in the *Church and religion* domain appear to level off, they keep slightly increasing in the *Clothing and personal hygiene* domain.

### Multiple Regression Over Distance Matrices

The first step of our analyses aimed to uncover how linguistic distance is influenced by several language-external factors: geographic distance, dialect area, the national border, separation by water, and differences in population size. The domain-based results are discussed below first, after which we present a summary of the findings across the four domains. As we focused on comparing MRM and LMER as analysis techniques, the discussion below only includes the results of the MRM analyses. However, we have included the Partial Mantel correlations—which showed exactly the same patterns—as well as the correlations between the predictor variables (which were all small to moderate, all r’s < 0.5) in our Supplementary Materials.

#### The *Church and Religion* Domain

Table 6 shows the MRM results for the *Church and religion* domain. Main factors that were correlated with linguistic distance were geographic distance, dialect area, the national border, and separation by water. In addition, there was a significant correlation with the interaction between separation by water and geographic distance. The language-external factors accounted for approximately 33% of the variation.

**TABLE 6:** Multiple regression over distance matrices (MRM) results for the *Church and religion* domain.

	Estimate	*P*
Intercept	0.820	<0.001
Log geographic distance	0.010	<0.001
Dialect area	0.003	0.009
National border	0.033	<0.001
Border * distance	−0.003	0.110
Separation by water	−0.023	0.001
Water * distance	0.008	<0.001
Log population difference	0.001	0.266

R^2^ = 0.329.

#### The *Clothing and Personal Hygiene* Domain

Table 7 shows the MRM results for the *Clothing and personal hygiene* domain. Main factors that were correlated with linguistic distance were geographic distance, dialect area, the national border, and separation by water. In addition, there were significant correlations with the interaction between the national border and geographic distance, and the interaction between separation by water and geographic distance. The language-external factors accounted for approximately 45% of the variation.

**TABLE 7:** Multiple regression over distance matrices (MRM) results for the *Clothing and personal hygiene* domain.

	Estimate	*p*
Intercept	0.698	<0.001
Log geographic distance	0.039	<0.001
Dialect area	0.004	0.004
National border	0.071	<0.001
Border * distance	−0.006	0.010
Separation by water	−0.022	0.003
Water * distance	0.005	0.015
Log population difference	0.000	0.987

R^2^ = 0.453.

#### The *Human body* Domain

Table 8 shows the MRM results for in the *Human body* domain. Main factors that were correlated with linguistic distance were geographic distance, dialect area, and the national border. There were no significant correlations with interactions between these factors. The language-external factors accounted for approximately 26% of the variation.

**TABLE 8:** Multiple regression over distance matrices (MRM) results for the *Human body* domain.

	Estimate	*p*
Intercept	0.769	<0.001
Log geographic distance	0.021	<0.001
Dialect area	0.004	0.006
National border	0.032	<0.001
Border * distance	−0.002	0.510
Separation by water	0.000	0.957
Water * distance	0.003	0.178
Log population difference	−0.001	0.393

R^2^ = 0.264.

#### The *Society and Education* Domain

Table 9 shows the MRM results for in the *Society and education* domain. The only main factor that was correlated with linguistic distance was geographic distance. There were no significant correlations with interactions between the factors. The language-external factors accounted for approximately 9% of the variation.

**TABLE 9:** Multiple regression over distance matrices (MRM) results for the *Society and education* domain.

	Estimate	*p*
Intercept	0.795	0.997
Log geographic distance	0.016	0.000
Dialect area	0.002	0.407
National border	0.011	0.374
Border * distance	0.004	0.197
Separation by water	−0.008	0.423
Water * distance	0.003	0.216
Log population difference	−0.001	0.263

R^2^ = 0.087.

#### Summary of the Four Domains

Table 10 summarizes the findings across the four semantic domains. The table lists which language-external factors significantly predicted linguistic distance in each domain (with “+” for positive coefficients and “−” for negative coefficients), indicates which of these was most strongly correlated based on the Partial Mantel correlations (shaded grey; see Supplementary Materials), and provides the R^2^-values obtained through the MRM analyses. The table shows that there are both similarities and differences across domains.

**TABLE 10:** Significant explanatory factors (+ for positive coefficients; − for negative coeffecients) and R^2^-values across the four semantic domains based on the multiple regression of distance matrices (MRM) analyses.

	Church and religion	Clothing and personal hygiene	Human body	Society and education
Log geographic distance	+	+	+	+
Dialect area	+	+	+	
National border	+	+	+	
Border * distance		−		
Separation by water	−	−		
Water * distance	+	+		
Log population difference				
MRM R^2^	0.33	0.45	0.26	0.09

Geographic distance significantly predicted linguistic distance in all four domains—Partial Mantel correlations ranged between r = 0.104 and r = 0.355; see Supplementary Materials. In fact, geographic distance was the strongest correlate with linguistic distance across all domains, which shows that linguistic differences within a relatively coherent dialect area such as the Limburgish one primarily arise from natural patterns of contact between communities.

Dialect area significantly predicted linguistic distance in three domains—not for the *Society and education* domain. As expected, linguistic distances were higher when locations are from different dialect areas, highlighting the role of smaller coherent subunits within an overall dialect area.

The national border significantly predicted linguistic distance in three of the four domains—again, not for the *Society and education* domain. The coefficients were always positive, confirming that the border acts as an additional barrier to contact between dialects. There was only one domain for which there was a significant interaction between the border and geographic distance, which indicates that the border generally acts as a barrier irrespective of distance. However, the fact that this interaction was negative seems to show that with increasing distance, the effect of the national border as a barrier can diminish. This was expected given that large distances between locations already hinder contact in themselves.

Separation by water significantly predicted linguistic distance in two of the four domains. Interestingly, the coefficients were always negative, indicating that dialects on separate sides of the Meuse river are more like each other. This finding might be counterintuitive at first, but in both cases, there was a positive significant interaction between separation by water and geographic distance, which we believe to be important in understanding this effect. The Meuse river provides an important means of transport in the area and upstream/downstream travel facilitates contact between towns on opposite sides even if they are relatively far apart. However, the further apart two locations are from each other, the more likely it is that neither of them is close to river at all, and the less likely it is that the Meuse facilitates contact between them, and so the positive interaction reverses its effect on linguistic distance.

In contrast to previous work that found population size to play an important role in linguistic variation (although see e.g., Nerbonne and Heeringa, 2007), difference in population size between the two locations was not significantly correlated with linguistic distance in any of the domains investigated in this study.

Finally, results from the MRM analyses showed that language external factors accounted for between only 9% and up to 45% of the variance, showing that the predictive power of such external factors can differ considerably between domains.

### Regression Analyses of Language-External Factors

In the second step of our analyses, we used linear mixed-effect modelling to further analyze the data. Critically, this approach allowed us to include location as a random variable in the analyses, thereby making it possible to account for differences in individual uniqueness of the locations included in our study. As with the previous analyses, we present the results on a domain basis, followed by a summary of the findings, and finally compare these results to what was found in the MRM analyses.

#### Language-External Factors in the *Church and Religion* Domain

Table 11 shows the results of the linear mixed-effect modelling for the *Church and religion* domain. Significant predictors of linguistic distance were geographic distance, dialect area, the national border, and separation by water. In addition, there were significant interaction effects between the national border and distance, as well as separation by water and distance. Overall, the model accounted for approximately 54% of the variance, of which 23% was accounted for by the inclusion of the random effect of location.

**TABLE 11:** Linear mixed-effect modelling results for the *Church and religion* domain, showing beta coefficients, standard errors, t-values and significance levels.

	*β*	SE	*t*	*p*
Intercept	−0.015	0.045	0.34	0.731
Log geographic distance	0.299	0.009	34.39	<0.001
Dialect area	0.022	0.007	2.92	0.003
National border	0.352	0.008	42.99	<0.001
Border * distance	0.034	0.008	4.06	<0.001
Separation by water	0.097	0.007	13.72	<0.001
Water * distance	0.035	0.008	4.46	<0.001
Log population difference	0.012	0.009	1.38	0.167

Conditional R^2^ = 0.538, Marginal R^2^ = 0.314.

#### Language-External Factors in the *Clothing and Personal Hygiene* Domain

Table 12 shows the results of the linear mixed-effect modelling for the *Clothing and personal hygiene* domain. Significant predictors of linguistic distance were geographic distance, dialect area, the national border, and separation by water. In addition, there were significant interaction effects between the national border and distance, as well as between separation by water and distance. Overall, the model accounted for approximately 55% of the variance, of which 13% was accounted for by the inclusion of the random effect of location.

**TABLE 12:** Linear mixed-effect modelling results for the *Clothing and personal hygiene* domain, showing beta coefficients, standard errors, t-values and significance levels.

	*β*	SE	*t*	*p*
Intercept	−0.036	0.027	1.33	0.183
Log geographic distance	0.460	0.005	86.59	<0.001
Dialect area	−0.014	0.004	3.22	0.001
National border	0.346	0.005	72.43	<0.001
Border * distance	0.136	0.005	25.60	<0.001
Separation by water	0.011	0.005	2.24	0.025
Water * distance	−0.020	0.005	3.99	<0.001
Log population difference	−0.003	0.006	0.46	0.645

Conditional R^2^ = 0.546, Marginal R^2^ = 0.420.

#### Language-External Factors in the *Human body* Domain

Table 13 shows the results of the linear mixed-effect modelling for the *Human body* domain. Significant predictors of linguistic distance were geographic distance, the national border, and separation by water. In addition, there were significant interaction effects between the national border and distance, as well as separation by water and distance. Overall, the model accounted for approximately 51% of the variance, of which 22% was accounted for by the inclusion of the random effect of location.

**TABLE 13:** Linear mixed-effect modeling results for the *Human body* domain, showing beta coefficients, standard errors, t-values and significance levels.

	*β*	SE	*t*	*p*
Intercept	−0.015	0.037	0.41	0.681
Log geographic distance	0.383	0.006	64.31	<0.001
Dialect area	0.007	0.005	1.43	0.154
National border	0.241	0.005	47.00	<0.001
Border * distance	0.073	0.005	13.25	<0.001
Separation by water	0.097	0.005	2.68	<0.001
Water * distance	−0.012	0.005	2.42	0.016
Log population difference	0.011	0.006	1.86	0.063

Conditional R^2^ = 0.510, Marginal R^2^ = 0.286.

#### Language-External Factors in the *Society and Education* Domain

Table 14 shows the results of the linear mixed-effect modelling for the *Society and education* domain. Significant predictors of linguistic distance were geographic distance, the national border, and population difference. In addition, there was a significant interaction effect between the national border and distance. Overall, the model accounted for approximately 29% of the variance, of which 12% was accounted for by the inclusion of the random effect of location.

**TABLE 14:** Linear mixed-effect modeling results for the *Society and education* domain, showing beta coefficients, standard errors, t-values and significance levels.

	*β*	SE	*t*	*p*
Intercept	−0.007	0.028	0.27	0.791
Log geographic distance	0.215	0.007	28.92	<0.001
Dialect area	0.008	0.006	1.33	0.183
National border	0.287	0.007	41.53	<0.001
Border * distance	0.024	0.007	3.24	0.001
Separation by water	0.007	0.006	1.08	0.280
Water * distance	0.004	0.007	0.64	0.522
Log population difference	0.028	0.008	3.67	<0.001

Conditional R^2^ = 0.292, Marginal R^2^ = 0.167.

#### Summary of Language-External Factors Across the Four Domains

Table 15 summarizes the findings across the four domains. The table lists which language-external factors were significant predictors of linguistic distance in each domain (with “+” for positive coefficients and “−” for negative coefficients), indicates which of these was the strongest predictors (shaded grey), and provides the conditional and marginal R^2^-values obtained through the linear mixed-effect modelling.

**TABLE 15:** Significant predictors across the four semantic domains (strongest predictor highlighted), and conditional and marginal R^2^-values for the linear mixed-effect models.

	Church and religion	Clothing and personal hygiene	Human body	Society and education
Log geographic distance	+	+	+	+
Dialect area	+	−		
National border	+	+	+	+
Border * distance	+	+	+	+
Separation by water	+	+	+	
Water * distance	+	−	−	
Log population difference				+
Conditional R^2^	0.54	0.55	0.51	0.29
Marginal R^2^	0.31	0.42	0.29	0.17

A conspicuous and reassuring outcome is the similarity of the MRM R^2^s in Table 10 and the Marginal R^2^s in Table 15. Both techniques approximately use the same sources of variation, but in the LMERs the random part (the random intercepts of the locations) is defined separately and excluded from the marginal R^2^. The conditional R^2^s are therefore higher, even much higher in our analyses, because of the relevance of individual dialect differences. At the same time, this has the consequence that we have more and stronger effects in the LMER analyses because they are related to the part of the variation defined as the conditional R^2^s.

As found in the correlational analyses, geographic distance was a significant predictor of linguistic distance in all domains. In contrast to the correlational analyses however, geographic distance was the strongest predictor in only two of the four domains. Interestingly, these were the two least standardized domains (*Clothing and personal hygiene*, and *the Human body*), highlighting that patterns of linguistic variation develop naturally through contact when there is no additional homogenization that results from standardization processes.

Differences between dialect areas significantly predicted overall linguistic distances in only two of the four semantic domains. That the effect in one of these domains (*Clothing and personal hygiene*) was negative is puzzling, but its small effect size indicates that the effect is negligible (*β* = −0.014).

The national border was a significant predictor of linguistic distance in all semantic domains, and it was the strongest predictor in two domains. These were the more standardized domains (*Church and religion*, and *Society and education*), which shows that overall standardization differences between Belgium and the Netherlands are reflected in increased linguistic differences between Limburgish varieties from different countries. Contrary to what we originally expected, this was also the case for the *Church and religion* domain. In addition, all domains showed a significant positive interaction between the national border and geographic distance, indicating that these two factors work in tandem with increased linguistic distances as a result.

In contrast to the results reported above (*Summary of the Four Domains*), the mixed-effect regressions showed increasing linguistic distances between locations that were separated by water—which is more in line with what we initially expected. That this effect was significant in combination with the national border indicates that they are independent barriers to contact. In addition, there was a significant interaction effect between separation by water and geographic distance in the three domains for which there was a main effect. In two (less standardized) domains, this interaction was negative indicating that the effect of separation by water decreases over distance, but for the *Church and religion* domain, the two work in tandem to further increase linguistic distances between locations.

Differences in population size turned out significant in only one domain, which is largely in line with the lack of significant correlations found in *Multiple Regression Over Distance Matrices*. The positive value of this effect seems to indicate that there are large linguistic distances between communities of different sizes.

Finally, R^2^-values of the mixed-effect models were on average around 20 percentage points higher than those for the MRM analyses. To assess the value of including location as a random variable, we compared the models presented above with models that did not include random effects, which showed that the random effect significantly improved the model for all domains (all *p*’s < 0.001). Including location as a random effect accounted for between 12 and 23 percentage points across the domains.

#### Spatial Patterns in Dialect Uniqueness

**FIGURE 6:**
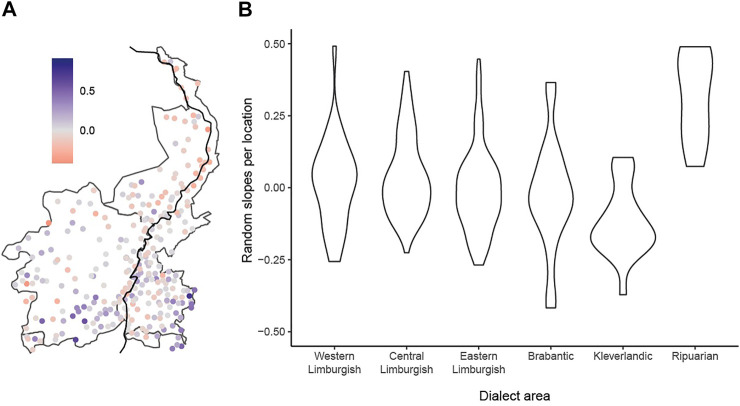
Map showing the Limburgish area with values of the mean random intercept for each location **(A)**, and distribution of random intercepts across six dialect areas **(B)**.

Dialect uniqueness has been addressed before, e.g., by Jeszenszky et al. (2019: 17), who provide a map of Japonic varieties showing average linguistic distance toward all other survey locations. While their use of average distance was able to pick up the mixed nature of varieties spoken in Hokkaido, the average linguistic distances in their map seem to be highest in peripheral areas, which is to be expected given they’re the furthest away from most other varieties. One way to better take this periphery component into consideration is to use the random intercepts of the LMER analysis. This makes it possible to further investigate spatial patterns in the random effect, i.e., spatial patterns in dialect uniqueness. To do so, we plotted all locations on a map of the Limburg area and colored them according to their mean random effect over the four semantic domains—see Figure 6, panel (a). In addition, we created a set of violin plots to show the distribution of the random intercepts across the six dialect areas—see Figure 6, panel (b).

As the figure shows, the random intercept for varieties in the three core areas (Western-, Central-, and Eastern Limburgish) center around zero and show a similar distribution in each area. The peripheral areas show more differences, however. While random intercepts for the locations in the Brabantic area show a pattern that is similar to the core Limburgish varieties, intercepts for the Kleverlandic varieties are skewed towards negative values (indicating smaller linguistic distances than expected), whereas intercepts for the Ripuarian varieties in the east, and some varieties in the southwest are skewed towards higher positives values (indicating larger linguistic differences than expected). For the Ripuarian data, these results are expected as the Ripuarian dialects are linguistically much closer to German dialects than the other dialects spoken in the Limburgish dialect area. Similarly, the varieties in the southwest have been shown to be influenced by French due to their proximity to the Germanic-Romance language border (van Hout, Kruijsen and Gerritsen, 2014), and this influence is particularly strong for the *Clothing and personal hygiene* domain (Franco et al., 2019a) included here. Finally, for the Kleverlandic dialects, the negative intercepts may perhaps be explained by the fact that these dialects are geographically the most outspoken edge of the Limburgish dialect area, where many very large geographical distances are expected to predict large linguistic distances. The negative intercepts seem to correct this peripheral overestimation as in e.g., Jeszenszky et al. (2019). Thus, the dialects that are spoken in the Kleverlandic region seem to resemble the language of the central regions more than expected on the basis of their location.

### Including Language-Internal Factors

Our final step of the analyses comprised the inclusion of language-internal factors to the linear mixed-effect model. As described above (see above, *Language-Internal Factors*), we coded several characteristics for each semantic domain: 1) the number of subsections at different levels of depth, 2) the number of concepts, both in total and at different levels of depth, 3) the ratio of multi-word concepts, and 4) the mean and median length of the concept headword.

As many of the language-internal factors were highly correlated (see Supplementary Table S10 for a complete overview), we conducted principal factor analysis to determine the structure of their common variance, which showed that our set of internal factors was computationally singular. As such, we merged all internal factors into a single variable based on the mean of their z-values.

We then conducted a series of linear mixed-effect regression analyses, which included all external factors as described in the previous section, with the addition of 1) semantic domain as a nominal variable (with *Society and education* as the reference level), 2) the single merged value for all internal factors combined, and 3) each language-internal factor individually. We compared these new models with the baseline model that only included language-external factors. A summary of these comparisons is shown in Table 16.

**TABLE 16:** Overview of models including language-internal factors, showing beta coefficients, Akaike information criterion values compared to the baseline model with external factors only, *χ* ^2^-values, and significance levels.

	*β*	AIC	*χ* ^2^	*p*
External factors only		1,82,438		
Domain (nominal)				
* Church and religion*	0.118	1,81,087	1,383	<0.001
* Clothing and personal hygiene*	0.079			
* Human body*	0.036			
All internal factors merged	0.069	1,81,920	528.7	<0.001
Subsections at one level of depth	0.068	1,81,928	521.5	<0.001
Subsections at two levels depth	0.020	1,82,409	42.24	<0.001
Subsections at maximum depth	−0.024	1,82,388	67.13	<0.001
Total number of concepts	0.035	1,82,331	129.1	<0.001
Concepts at one level of depth	−0.006	1,82,452	4.31	0.038
Concepts at two levels depth	0.039	1,82,297	156.5	<0.001
Concepts at maximum depth	0.076	1,81,834	618.1	<0.001
Ratio of multi-word concepts	0.057	1,82,086	359.3	<0.001
Mean concept length	0.085	1,81,623	828.0	<0.001
Median concept length	0.078	1,81,764	686.5	<0.001

df for Domain as nominal variable = 3; all other df’s = 1.

As the table shows, all individual language-internal factors significantly improved the model, indicating that there is added value in including language-internal factors when trying to model patterns of linguistic variation. For the individual factors, the two measures of concept headword length provided largest improvement—even more so than the merged value for all language-internal factors combined. Their positive betas confirm that for less salient concepts, larger linguistic distances are found across the Limburgish dialect area. At the same time however, the AIC values show that the addition of semantic domain as a nominal variable produces the best model, suggesting that there are additional domain-specific characteristics that were not captured by the language-internal factors here.

## Discussion

In this paper, we showcased spatial analysis techniques for dialect geography. After conducting a correlational analysis with Mantel correlations and MRM, we used linear mixed-effects regression (LMER) modelling to further investigate the effect of language-external factors while accounting for location-based variation by including it as a random factor. This method also allowed us to critically assess the importance of a set of independent variables that have been shown to affect processes of linguistics diffusion, both language-external and language-internal. All in all, our results confirm that geographic distance is a very important predictor of linguistic differences. However, depending on the semantic domain under scrutiny, other language-external factors were shown to play a significant role as well. For example, in the semantic domains of *Church and religion* and *Society and education*, our analyses revealed that the national border has a larger effect. Finally, our models improved when language-internal variables were included in the analysis, further confirming that linguistic distances differ between semantic domains, an observation that may be relevant for future work in lexical dialectology, as well as for lexical research more broadly.

There are a number of advantages of using the techniques proposed here over methods that have a longer standing in the field. First, comparing the results for the external factors obtained with MRM vis-à-vis our linear mixed-effects models shows that they are highly similar across the board, indicating that our LMER approach is a suitable technique for this line of research. Moreover, mixed-effects modelling makes it possible to incorporate the inherent uniqueness of individual locations by handling them as a random factor. While previous work has used individual locations as random effects (e.g., Wieling, 2012; Wieling et al., 2014; Wieling et al., 2018), these studies compared each location to only a single point of reference, and the random effect gives insight into how each dialect compares to the other dialects in its divergence from the standard. In contrast, the LMER approach described here uses linear models through which we can apply regression to *all* pairs of linguistic differences. As such, each location is compared with all other locations and the random effect provides the additional insight into the individual uniqueness of each dialect. In this study specifically, where we aimed to understand the relationship between linguistic and geographic distance, the use of random intercepts informs us of the position of individual locations (or groups of locations) in the overall linguistic area. While both correlational analyses also explore sources of variation, the random intercepts of locations in the LMERs are defined separately. The consequence of this is stronger and more clearly defined effects in the LMER analyses. Of course, another source of variation may be that, depending on the location, linguistic distances may increase at a higher or lower rate. Although we also examined the effect of such random slopes in the analyses, the models did not converge. We certainly need to further explore the many possibilities of linear mixed regression.

The LMER method proposed here shares with previous approaches (GAM; e.g., Wieling, 2012) the opportunity of incorporating data that cannot be coded into pairwise distance matrices, such as language-internal factors. This makes the method similar to other work in lectometry interested in the causes of variation in aggregate measures (Schneider, 1988; Pickl, 2013; Ruette and Speelman, 2013; Plevoets, 2020). Moreover, we might go even further and include the individual concepts as random factors (as in work using GAM), much as current approaches in psycholinguistic research (cf. Winter, 2019 for an introduction). In sum, the technique has large flexibility in handling random structures inherent to large data sets, allowing the researcher to systematically investigate latent sources of variation in their data. This is particularly relevant given the known importance of language-internal variables and general cognitive principles in linguistic variation (see the references in Franco et al., 2019b).

Some questions remain, however. First, our analyses used the Weighted Dissimilarity Value as a measure of linguistic distance, as its results were more regular than what we obtained for string edit distance. It is possible that the large number of data points, as well as long responses for a subset of concepts, may have resulted in large linguistic distances that contain unnecessary noise. More detailed investigation is needed to determine whether the use of string edit distance—as is common in dialectometry—becomes unstable in certain cases, e.g., for large datasets. Thus, follow-up research should investigate other measures for linguistic distance. One option worth exploring is weighing linguistic dissimilarity based on the geographic density of the lexical variants. Variants with a dense geographical distribution may prove more informative on the role of geography.

The current study uses straight line distances, but there are of course other ways of operationalizing geographic distance that have been used in studies on patterns of linguistic variation, such as travel distance (e.g., Inoue, 2004; Jeszenszky et al., 2019) and travel time (e.g., Gooskens, 2005; Jeszenszky et al., 2019), or using longitude/latitude (e.g., Wieling, 2012; Wieling et al., 2014; Wieling et al., 2018). For the Limburgish dialect area, we expect our results to stay the same when using such measures, as there are no other major geographical obstacles (e.g., mountain ranges, marshlands) that would further impede travel. For the whole of the Netherlands, straight line distance and travel distance have been shown to correlate strongly (r > 0.9; van Gemert, 2002). In fact, even in an area as mountainous as Japan, hiking distance and modern travel distance both correlate strongly with straight line distance (Jeszenszky et al., 2019). Nevertheless, Gooskens (2005) showed that incorporating *historical*—rather than modern—travel times produced better models for Norwegian varieties, so there are potential benefits for some linguistic areas provided that such historical data is available.

Another open question concerns the identification and importance of language-internal factors. While we were able to show that inclusion of language-internal factors improved our model, the largest improvement was obtained by simply including semantic domain as a nominal variable. It thus remains an open question which factors cause semantic domains to differ from each other. While we specifically chose four semantic domains that vary with regard to their degree of standardization and cultural variability, these interpretations do not unequivocally explain the results we obtained. For example, Table 16 shows that, if language-external variables are controlled for, the *β* is the highest in the field C*hurch and religion*, which is the field where little variation would be expected. Further work looking at additional domains and subdomains is needed to better understand the role of different language-internal factors in the emergence and persistence of variation across a dialect area.

The approach taken in the current study might be qualified as *computational dialect geography*. In fact, we prefer this label over the one of *dialectometry*. Dialectometry perfectly fits the developments in the past, i.e., stipulating that dialect phenomena are measurable, but as new computational procedures and algorithms emerge and get applied, we believe there is a broader potential of handling and analyzing language variation data, including the many internal and external factors, giving the floor to computational dialect geography, i.e., computational sociolinguistics. These developments give room to additional next-level techniques such as machine learning and deep learning to research dialect classification problems, and computational intelligence to understand the trade-off between processes of convergence and divergence in short-term and long-term communicative processes. Future work can also build on methods that are used to optimize complex functions to better understand the functional relation between linguistic and geographic distance. Finally, the use of simulations in predicting linguistic variation is not new (see e.g., Hard, 1972), but incorporating techniques from other fields can move these attempts forward in testing and revealing the underlying parameters and processes of linguistic variation.

## Data Availability

The datasets presented in this study can be found in online repositories. The names of the repository/repositories and accession number(s) can be found below: https://osf.io/2jcxa/.

## Footnotes

1 The most important questionnaire used for this semantic field was distributed across the dialect area in 1989.

2 The data was collected in March 2019.

3 Available at https://www.wikipedia.org/. Wikipedia is of course not an ideal source for this type of information, but we believe there is merit in this case because the pages devoted to neighborhoods or districts within an administrative community often display a large degree of local pride identity, e.g., by naming famous people from the community or by describing its history and local traditions. This makes it unlikely that incorrect information will be added to the website. In addition, every Wikipedia article also has a “Talk page”, which is used as a discussion board to improve the article (e.g., by correcting faulty information). In total, for 155 out of 660 communities, Wikipedia data was used. The Wikipedia data is mostly based on domicile information and typically describes the number of residents for a particular year, most often between 2006 and 2018.

4 The models that will be discussed below, include locations as random intercepts. Although we also built models with random slopes, these models did not converge.
