# Supplementary material for: Linking Linguistic and Geographic Distance in Four Semantic Domains: Computational Geo-Analyses of Internal and External Factors in a Dialect Continuum
Source: Front Artif Intell. 2021 Jun 4;4:668035. doi: 10.3389/frai.2021.668035 (PMC8211982; doi:10.3389/frai.2021.668035)
Supplement: Supplementary file 1 [file DataSheet1.docx]

Supplementary Material

# Simulation study comparing the effectiveness of Mantel correlations, Multiple Regression over distance Matrices (MRM) and linear mixed-effect regression (LMER)

To further test the added value of linear mixed-effects regression (LMER) in comparison to Mantel correlations and Multiple Regression on distance Matrices (MRM) analyses, we simulated linguistic distances in a dialect area split in two by a national border. The simulation allows for tight control over the strength of various predictors and thus makes it possible to determine the effectiveness of the different analysis methods.

**Creating the dialect area**

We simulated a dialect area consisting of 25 locations arranged in a 5*5 grid, spaced apart at intervals of 10 units on both axes. We added a randomly generated unit (normally distributed around a mean of *M* = 0 and a standard deviation of *SD* = 2), to both the x-coordinate (ranging between 0 and 60) and the y-coordinate (ranging between 0 and 60) of each location to add some variation in the distance between locations. Next, we generated a random border through the dialect area. This border consisted one horizontal and two vertical segments in the shape shown in Supplementary Figure 1. The *y*-value for the horizontal segment ranged between 25 and 35, whereas the *x*-values for the vertical segments ranged between 15 and 30, and 25 and 40, respectively. Each location was then assigned to either country “A” (when located left of the border), or country “B” (when located right of the border)—see Supplementary Figure 1.


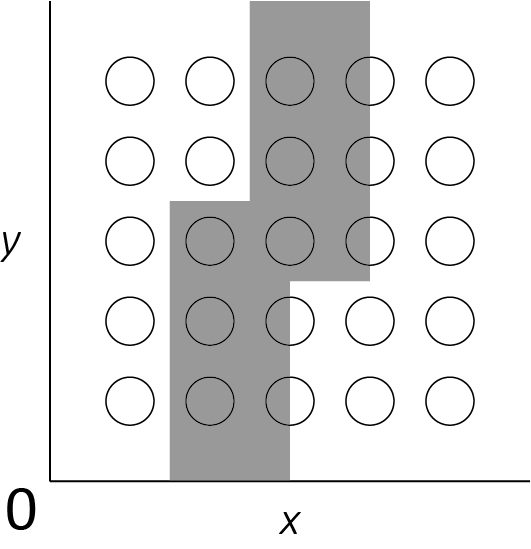


Supplementary Figure 1. Map of the simulated dialect area with circles indicating 99.7% confidence intervals of the x and y coordinates of the location, and the shaded area indicating the possible range of the border running through the area.

Then, we simulated a range of population sizes across the 25 locations. To do this, we first randomly assigned between 1 and 10 initial “citizens” to each location. Each citizen received an id according to which location they were assigned to. Then we increased the population of each location through a process akin to preferential attachment. We generated one new citizen at a time and assigned it a location id, which was randomly drawn from all existing location ids at that step. Through this method, locations with a higher population had a higher chance of being picked again, which creates a range of population sizes across the 25 locations. This stepwise process continued until the population of all locations combined reached 100,000. Supplementary Figure 2 shows the results of a complete run of this simulation.


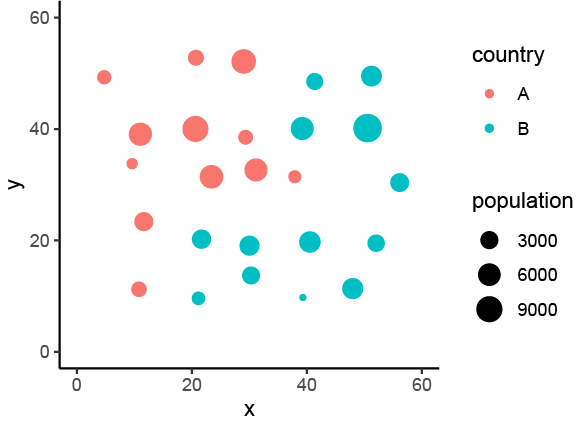


Supplementary Figure 2. Three-step simulation of a dialect area consisting of 25 locations across two different countries. In the first step, we generated x-coordinates and y-coordinates for each location based on a 5*5 grid. In the second step, we generated a random national border and assigned each location to either country “A” or “B”. In the final step, we generated population sizes for all locations.

**Simulating linguistic distances**

After generating the dialect area, we computed linguistic distances between all locations. We used the following principles to do this:

- Following the process of isolation by dispersal limitation (IBDL; Wright 1943), the amount of contact between locations is inversely proportional to the distance between them.
- Following Trudgill's (1974) gravity model of diffusion, the intensity of contact between locations is proportional to their relative sizes, and inversely proportional to the distance between them.
- The border creates a barrier that hinders contact between locations on opposite sides.

Using these principles, the basic formula for linguistic distance (*d*) we used was:

$$d = c_{1}*distance - c_{2}*contact intensity + c_{3}*separation by border$$

For *distance*, we used the Euclidean distance between each pair of locations. For *contact intensity* we used the natural logarithm of the value produced by Trudgill’s (1974, p. 233) gravity formula. For *separation by border* we used either 0 (for locations on the same side), or 1 (for locations on opposite sides).

In addition, our basic formula contains three constants (*c_1_*, *c_2_*, and *c_3_*) which can be varied to increase or decrease the influence of each respective factor. In the simulation presented here, we set *c_1_* to 0.01, *c_2_* to 0.01, and *c_3_* to 0.1.

**Introducing noise to the linguistic distance values**

The formula above produces perfectly predictable linguistic distances and is therefore not suitable for testing analysis methods. So, we added random noise to the linguistic distance values. We distinguished two types of noise:

- We added random noise added to each language pair, intended to represent the variable nature of language.
- We added a specific random value assigned to each location, intended to represent the uniqueness of each individual dialect.

We varied the amount of both types of noise to test the following hypotheses:

- Increased noise reduces the overall predictability of linguistic distances, which should be reflected in lower r values in the partial Mantel correlations and lower R^2^ values in MRM and LMER.
- For increased random noise, MRM and LMER analyses should perform equally well.
- For increased dialect uniqueness values, the inclusion of locations as random effects in LMER analyses means they should outperform MRM.

Supplementary Table 1 shows the results of varying levels of random noise. As expected, increased noise leads to lower correlations and lower R^2^ values overall. Mantel analyses and LMER analyses were affected to a similar extent, but both estimated the effects of distance (set at 0.01) and country (set at 0.1) accurately. Predictably, the correlation between the random effect in the LMER model and dialect uniqueness decreased with more random noise.

Supplementary Table 2 shows the results of varying levels of dialect uniqueness. As expected, increased uniqueness leads to lower correlations and lower R^2^-values overall. This time however, Mantel analyses were more strongly affected than LMER analyses—for the highest level of dialect uniqueness, the MRM analysis’ R^2^ was .52, whereas the LMER analysis’ R^2^ was .68. Predictably, the correlation between the random effect in the LMER model and dialect uniqueness increased with higher levels of uniqueness, indicating that the inclusion of a random effect can accurately account of this uniqueness.

| Supplementary Table 1.  Results of three analysis methods with dialect uniqueness constant at 0.01 and varying levels of random noise | | | | |
| --- | --- | --- | --- | --- |
|  |  | Amount of random noise | | |
|  |  | 0.01 | 0.05 | 0.1 |
| Partial Mantel  (correlations) | Geographic distance | r = .97 | r = .91 | r = .76 |
|  | Separation by border | r = .86 | r = .65 | r = .48 |
| MRM analysis  (coefficients) | Geographic distance | 0.011 | 0.011 | 0.01 |
|  | Separation by border | 0.095 | 0.098 | 0.11 |
|  | R^2^ | .97 | .88 | .71 |
| Mixed-effect model (coefficients) | Geographic distance | 0.010 | 0.010 | 0.010 |
|  | Separation by border | 0.096 | 0.097 | 0.099 |
|  | marginal R^2^ | .96 | .88 | .72 |
|  | conditional R^2^ | .97 | .89 | .73 |
|  | Correlation between the random effect and dialect uniqueness | r = .84 | r = .78 | r = .53 |

| Supplementary Table 2.  Results of three analysis methods with random noise constant at 0.01 and varying levels of dialect uniqueness | | | | |
| --- | --- | --- | --- | --- |
|  |  | Dialect uniqueness | | |
|  |  | 0.01 | 0.05 | 0.1 |
| Partial Mantel  (correlations) | Geographic distance | r = .97 | r = .84 | r = .64 |
|  | Separation by border | r = .86 | r = .49 | r = .27 |
| MRM analysis  (coefficients) | Geographic distance | 0.011 | 0.011 | 0.009 |
|  | Separation by border | 0.095 | 0.093 | 0.074 |
|  | R^2^ | .97 | .80 | .52 |
| Mixed-effect model (coefficients) | Geographic distance | 0.010 | 0.010 | 0.008 |
|  | Separation by border | 0.096 | 0.092 | 0.076 |
|  | marginal R^2^ | .96 | .80 | .50 |
|  | conditional R^2^ | .97 | .88 | .68 |
|  | Correlation between the random effect and dialect uniqueness | r = .84 | r = .96 | r =.97 |

# Dialect classification of the locations in the database

Supplementary Table 3 lists all locations included in the database, their classification into one of six dialect areas: the three core areas *Western Limburgish*, *Central Limburgish*, and *Eastern Limburgish*, and the three peripheral areas *Brabantic*, *Kleverlandic*, and *Ripuarian*, and for which semantic domains they have data included. Supplementary Figure 1 is a map of all locations, color-coded by their classification.

| Supplementary Table 3. List of locations in the database, their classification and data availability per domain. | | | | | |
| --- | --- | --- | --- | --- | --- |
|  |  | Domain | | | |
| Location | Dialect area | Church and religion | Clothing and personal hygiene | Human body | Society and education |
| Achel | Western Limburgish | x | x | x | x |
| Afferden | Kleverlandic |  |  |  | x |
| Alken | Western Limburgish |  |  | x | x |
| Amby | Eastern Limburgish | x |  |  |  |
| America | Kleverlandic |  | x |  |  |
| Amstenrade | Eastern Limburgish | x | x | x |  |
| As | Central Limburgish | x | x | x | x |
| Baarlo | Eastern Limburgish | x | x | x | x |
| Baexem | Eastern Limburgish |  |  |  | x |
| Beegden | Eastern Limburgish | x |  |  | x |
| Beek | Eastern Limburgish | x | x | x |  |
| Beesel | Eastern Limburgish | x | x |  |  |
| Belfeld | Eastern Limburgish |  |  | x | x |
| Bemelen | Eastern Limburgish |  |  | x |  |
| Berg | Central Limburgish |  |  |  | x |
| Bergen | Kleverlandic |  |  | x |  |
| Beringen | Western Limburgish |  |  | x |  |
| Berverlo | Western Limburgish |  |  | x | x |
| Beverst | Central Limburgish |  |  |  | x |
| Bilzen | Central Limburgish |  |  | x | x |
| Bleijerheide | Ripuarian |  |  | x |  |
| Blerick | Eastern Limburgish | x | x | x | x |
| Blitterswijck | Kleverlandic |  | x |  |  |
| Bocholt | Central Limburgish | x | x | x | x |
| Bocholtz | Ripuarian |  | x | x | x |
| Boekend | Eastern Limburgish |  |  | x | x |
| Boeket | Central Limburgish |  |  | x | x |
| Boekt Heikant | Western Limburgish | x |  | x |  |
| Bokrijk | Western Limburgish |  |  |  | x |
| Boorsem | Central Limburgish |  | x | x |  |
| Borgharen | Central Limburgish |  |  | x |  |
| Borgloon | Western Limburgish | x |  | x |  |
| Borlo | Brabantic |  |  | x |  |
| Born | Central Limburgish | x |  | x |  |
| Boshoven | Central Limburgish | x |  | x |  |
| Boukol | Eastern Limburgish |  |  |  | x |
| Bree | Central Limburgish | x | x | x | x |
| Broekhuizen | Kleverlandic |  |  | x | x |
| Brunssum | Eastern Limburgish | x |  | x | x |
| Brustem | Brabantic |  |  | x |  |
| Buchten | Central Limburgish |  |  | x | x |
| Bunde | Central Limburgish | x |  |  | x |
| Chevremont | Ripuarian |  | x | x |  |
| Diepenbeek | Central Limburgish | x | x | x | x |
| Dilsen | Central Limburgish |  |  | x |  |
| Doenrade | Eastern Limburgish | x | x | x | x |
| Donk | Brabantic |  |  | x |  |
| Echt | Central Limburgish | x | x | x | x |
| Egchel | Eastern Limburgish | x |  | x |  |
| Eigenbilzen | Central Limburgish | x | x | x | x |
| Eijsden | Central Limburgish | x |  | x |  |
| Eind | Central Limburgish |  |  | x |  |
| Einighausen | Eastern Limburgish |  |  | x |  |
| Eisden | Central Limburgish |  | x | x |  |
| Eksel | Western Limburgish | x | x | x | x |
| Ell | Central Limburgish | x | x | x | x |
| Elsloo | Central Limburgish |  |  |  | x |
| Epen | Eastern Limburgish | x | x |  |  |
| Eygelshoven | Ripuarian |  |  |  | x |
| Eys | Eastern Limburgish | x | x |  | x |
| Geistingen | Central Limburgish |  | x |  | x |
| Geleen | Eastern Limburgish | x | x | x | x |
| Gelieren Bret | Central Limburgish | x |  |  |  |
| Gemmenich | Ripuarian |  |  |  | x |
| Genk | Central Limburgish |  |  | x | x |
| Gennep | Kleverlandic | x | x |  | x |
| Geulle | Central Limburgish | x | x | x | x |
| Gingelom | Brabantic |  |  |  | x |
| Gors Opleeuw | Western Limburgish |  |  |  | x |
| Grathem | Central Limburgish | x |  | x |  |
| Grazen | Brabantic |  |  | x |  |
| Grevenbicht / Papenhoven | Central Limburgish | x | x |  | x |
| Gronsveld | Eastern Limburgish | x |  | x | x |
| Grote Spouwen | Central Limburgish |  |  | x |  |
| Gulpen | Eastern Limburgish | x | x |  | x |
| Guttecoven | Central Limburgish | x | x | x | x |
| Haelen | Eastern Limburgish | x |  | x | x |
| Halen | Brabantic | x |  | x | x |
| Haler | Central Limburgish | x | x |  |  |
| Hamont | Western Limburgish | x |  | x | x |
| Hasselt | Western Limburgish | x | x | x | x |
| Hechtel | Western Limburgish |  | x | x | x |
| Heel | Eastern Limburgish | x | x |  | x |
| Heerlen | Eastern Limburgish | x |  | x | x |
| Heerlerheide | Eastern Limburgish |  |  | x | x |
| Heers | Western Limburgish |  | x |  |  |
| Hees | Central Limburgish |  |  |  | x |
| Herk De Stad | Brabantic | x |  | x |  |
| Herten | Eastern Limburgish | x |  | x | x |
| Heugem | Central Limburgish | x | x | x | x |
| Heythuysen | Eastern Limburgish | x |  | x | x |
| Hoensbroek | Eastern Limburgish | x | x | x | x |
| Hoepertingen | Western Limburgish |  | x | x | x |
| Hoeselt | Central Limburgish | x | x | x | x |
| Holtum | Central Limburgish | x | x | x | x |
| Hopmaal | Western Limburgish |  |  | x |  |
| Horn | Eastern Limburgish |  | x | x | x |
| Horst | Kleverlandic | x |  | x | x |
| Hout Blerick | Eastern Limburgish |  |  | x |  |
| Houthalen | Western Limburgish | x | x | x | x |
| Hulsberg | Eastern Limburgish | x |  |  |  |
| Ingber | Eastern Limburgish | x |  |  |  |
| Itteren | Central Limburgish | x |  |  |  |
| Ittervoort | Central Limburgish | x |  | x | x |
| Jabeek | Eastern Limburgish | x |  | x |  |
| Jeuk | Brabantic | x | x | x | x |
| Kanne | Central Limburgish |  |  | x | x |
| Kapel in t Zand | Eastern Limburgish | x |  |  | x |
| Kaulille | Western Limburgish | x |  | x | x |
| Kelmis | Ripuarian |  |  |  | x |
| Kelpen | Central Limburgish | x | x |  |  |
| Kerensheide | Central Limburgish |  |  | x | x |
| Kerkhoven | Brabantic |  |  |  | x |
| Kerkrade | Ripuarian | x | x | x | x |
| Kermt | Western Limburgish | x |  | x |  |
| Kessel | Eastern Limburgish |  | x |  |  |
| Kesseleik | Eastern Limburgish | x | x |  |  |
| Ketsingen | Central Limburgish |  |  | x |  |
| Kinrooi | Central Limburgish | x |  | x | x |
| Klimmen | Eastern Limburgish | x | x | x | x |
| Koersel | Western Limburgish |  |  |  | x |
| Koningsbosch | Eastern Limburgish |  | x |  |  |
| Kunrade | Eastern Limburgish |  | x |  |  |
| Kwaadmechelen | Brabantic |  |  | x | x |
| Lanaken | Central Limburgish |  |  | x |  |
| Lanklaar | Central Limburgish | x |  | x | x |
| Lauw | Central Limburgish |  |  |  | x |
| Leopoldsburg | Brabantic | x |  | x | x |
| Leuken | Central Limburgish | x |  |  | x |
| Leveroij | Central Limburgish |  |  |  | x |
| Limbricht | Eastern Limburgish | x |  | x | x |
| Linkhout | Brabantic |  |  | x |  |
| Linne | Eastern Limburgish |  | x |  |  |
| Loksbergen | Brabantic | x | x | x | x |
| Lommel | Brabantic | x | x | x | x |
| Lummen | Western Limburgish | x |  | x |  |
| Lutterade | Eastern Limburgish | x | x | x | x |
| Maasbracht | Central Limburgish | x |  | x | x |
| Maasbree | Kleverlandic | x | x | x | x |
| Maaseik | Central Limburgish | x |  | x | x |
| Maasmechelen | Central Limburgish | x | x | x | x |
| Maasniel | Eastern Limburgish | x |  | x | x |
| Maastricht | Central Limburgish | x | x | x | x |
| Mal | Central Limburgish |  |  | x |  |
| Margraten | Eastern Limburgish |  |  | x |  |
| Mechelen | Eastern Limburgish | x |  | x | x |
| Meerlo | Kleverlandic | x |  |  |  |
| Meers | Central Limburgish |  |  |  | x |
| Meerssen | Eastern Limburgish |  | x | x | x |
| Meeswijk | Central Limburgish | x |  |  | x |
| Meeuwen | Central Limburgish | x |  | x | x |
| Meijel | Kleverlandic | x | x | x | x |
| Melick | Eastern Limburgish | x | x |  |  |
| Membruggen | Central Limburgish |  | x |  |  |
| Merkelbeek | Eastern Limburgish | x |  |  |  |
| Merselo | Kleverlandic |  | x |  |  |
| Mesch | Eastern Limburgish |  |  | x | x |
| Mheer | Eastern Limburgish | x |  | x | x |
| Middelaar | Kleverlandic | x |  | x | x |
| Millen | Central Limburgish |  |  | x |  |
| Milsbeek | Kleverlandic | x |  | x |  |
| Montfort | Eastern Limburgish | x | x |  | x |
| Montzen | Eastern Limburgish |  | x | x | x |
| Moresnet | Eastern Limburgish |  |  |  | x |
| Munsterbilzen | Central Limburgish |  |  | x |  |
| Munstergeleen | Eastern Limburgish |  |  | x |  |
| Nederweert | Central Limburgish |  |  |  | x |
| Neer | Eastern Limburgish | x | x | x | x |
| Neerbeek | Eastern Limburgish |  | x |  | x |
| Neerharen | Central Limburgish | x |  | x |  |
| Neeritter | Central Limburgish | x |  | x | x |
| Neeroeteren | Central Limburgish |  |  | x | x |
| Neerpelt | Western Limburgish | x | x | x | x |
| Niel Bij Sint Truiden | Brabantic | x |  |  |  |
| Nieuwenhagen | Eastern Limburgish | x | x | x | x |
| Nieuwstadt | Eastern Limburgish | x | x | x |  |
| Noorbeek | Eastern Limburgish | x | x | x |  |
| Nunhem | Eastern Limburgish | x |  | x | x |
| Nuth | Eastern Limburgish | x | x | x | x |
| Obbicht | Central Limburgish | x | x |  | x |
| Oirlo | Kleverlandic | x | x | x | x |
| Oirsbeek | Eastern Limburgish | x |  | x | x |
| Oost Maarland | Central Limburgish | x |  | x | x |
| Opglabbeek | Central Limburgish | x | x | x | x |
| Opheers | Western Limburgish | x |  | x | x |
| Ophoven | Central Limburgish | x | x | x | x |
| Opoeteren | Central Limburgish |  | x |  |  |
| Ospel | Central Limburgish | x | x |  |  |
| Ottersum | Kleverlandic | x |  | x | x |
| Oud Caberg | Central Limburgish | x |  | x | x |
| Oud Waterschei | Central Limburgish | x |  |  |  |
| Overpelt | Western Limburgish | x |  |  | x |
| Paal | Western Limburgish |  |  | x | x |
| Panningen | Eastern Limburgish | x |  | x | x |
| Peer | Western Limburgish | x | x | x | x |
| Peij | Eastern Limburgish | x |  |  |  |
| Posterholt | Eastern Limburgish | x | x | x | x |
| Puth | Eastern Limburgish | x |  | x | x |
| Ransdaal | Eastern Limburgish |  |  | x |  |
| Rekem | Central Limburgish |  |  | x | x |
| Reuver | Eastern Limburgish | x | x | x | x |
| Riksingen | Central Limburgish |  |  | x |  |
| Rimburg | Eastern Limburgish |  |  |  | x |
| Roermond | Eastern Limburgish | x | x | x | x |
| Roggel | Eastern Limburgish |  | x | x |  |
| Romershoven | Central Limburgish |  |  | x |  |
| Roosteren | Central Limburgish | x |  |  | x |
| Rosmeer | Central Limburgish | x |  | x |  |
| Rotem | Central Limburgish | x |  | x |  |
| Rothem | Eastern Limburgish | x |  | x | x |
| Rukker | Eastern Limburgish | x |  |  |  |
| Rummen | Brabantic | x |  | x |  |
| S Gravenvoeren | Eastern Limburgish |  |  | x |  |
| Schaesberg | Eastern Limburgish | x |  |  | x |
| Schimmert | Eastern Limburgish | x | x | x | x |
| Schinnen | Eastern Limburgish | x | x | x | x |
| Schinveld | Eastern Limburgish |  |  | x | x |
| Schulen | Western Limburgish |  |  | x |  |
| Sevenum | Kleverlandic | x | x | x | x |
| Siebengewald | Kleverlandic |  | x |  |  |
| Simpelveld | Ripuarian | x |  |  |  |
| Sint Huibrechts Lille | Western Limburgish |  | x |  |  |
| Sint Martens Voeren | Eastern Limburgish |  | x |  |  |
| Sint Pieter | Central Limburgish |  |  |  | x |
| Sint Truiden | Brabantic | x | x | x | x |
| Sittard | Eastern Limburgish | x | x | x | x |
| Smeermaas | Central Limburgish | x |  |  | x |
| Spalbeek | Western Limburgish | x |  | x |  |
| Stein | Central Limburgish | x |  | x | x |
| Stevensweert | Central Limburgish |  |  | x | x |
| Stokkem | Central Limburgish | x | x | x |  |
| Susteren | Eastern Limburgish | x |  | x | x |
| Swalmen | Eastern Limburgish | x | x | x | x |
| Sweikhuizen | Eastern Limburgish |  | x |  |  |
| Tegelen | Eastern Limburgish | x | x | x | x |
| Ten Esschen | Eastern Limburgish | x |  |  | x |
| Terlinden | Eastern Limburgish | x | x |  |  |
| Tessenderlo | Brabantic |  | x | x | x |
| Teuven | Eastern Limburgish | x |  | x |  |
| Thorn | Central Limburgish | x | x | x | x |
| Tienray | Kleverlandic | x | x |  |  |
| Tongeren | Central Limburgish | x | x | x | x |
| Tungelroy | Central Limburgish | x | x | x | x |
| Ubachsberg | Eastern Limburgish |  | x |  | x |
| Uikhoven | Central Limburgish |  | x |  |  |
| Ulestraten | Eastern Limburgish | x | x | x | x |
| Urmond | Central Limburgish | x | x | x | x |
| Vaals | Ripuarian | x |  |  | x |
| Val Meer | Central Limburgish | x |  | x | x |
| Valkenburg | Eastern Limburgish | x | x | x | x |
| Velden | Eastern Limburgish | x |  |  | x |
| Veldwezelt | Central Limburgish |  |  | x | x |
| Velm | Brabantic | x |  | x |  |
| Venlo | Eastern Limburgish | x | x | x | x |
| Venray | Kleverlandic | x | x | x | x |
| Vijlen | Eastern Limburgish | x | x |  |  |
| Vliermaal | Central Limburgish |  |  | x | x |
| Vlijtingen | Central Limburgish | x |  |  |  |
| Vlodrop | Eastern Limburgish | x | x | x | x |
| Voerendaal | Eastern Limburgish | x | x |  | x |
| Vorsen | Brabantic |  |  | x | x |
| Vrusschemig | Eastern Limburgish |  |  |  | x |
| Wanssum | Kleverlandic |  |  |  | x |
| Waterloos | Central Limburgish | x |  |  |  |
| Waubach | Eastern Limburgish | x | x | x | x |
| Weert | Central Limburgish | x | x | x | x |
| Wellen | Western Limburgish | x |  | x | x |
| Welten | Eastern Limburgish | x |  |  |  |
| Wijlre | Eastern Limburgish | x |  |  |  |
| Wijnandsrade | Eastern Limburgish | x |  |  |  |
| Wintershoven | Central Limburgish | x |  | x |  |
| Wolder / Oud Vroenhoven / Wiler | Central Limburgish | x |  |  | x |
| Wyck | Central Limburgish | x | x | x | x |
| Zelem | Brabantic | x |  | x | x |
| Zichen Zussen Bolder | Central Limburgish | x |  | x |  |
| Zolder | Western Limburgish | x |  | x | x |
| Zonhoven | Western Limburgish | x | x | x | x |
| Zutendaal | Central Limburgish |  |  | x | x |

# Correlations between language-external factors across the four semantic domains

We calculated, for each semantic domain separately, Mantel correlations between the language-external factors using the *mantel* function (with 10,000 permutations and 1,000 bootstrap iterations on 95% confidence intervals) in the *ecodist* package (Goslee & Urban 2007) in R. Supplementary Table 4 shows the correlations for the *Church and religion* domain, Supplementary Table 5 the correlations for the *Clothing and personal hygiene* domain, Supplementary Table 6 for the *Human body* domain, and Supplementary Table 7 for the *Society and education* domain. Associations between the external factors were small to moderate, but as the tables shows, associations with population difference were negligible across all domains.

| Supplementary Table 4. Mantel correlations between language-external factors for the *Church and religion* domain. | | | | |
| --- | --- | --- | --- | --- |
|  | Dialect area | National border | Separation by water | Population difference |
| Log geographic distance | .413 | .283 | .256 | .087 |
| Dialect area |  | .298 | .261 | .050 |
| National border |  |  | .275 | .072 |
| Separation by water |  |  |  | -.009 |

| Supplementary Table 5. Mantel correlations between language-external factors for the *Clothing and personal hygiene* domain. | | | | |
| --- | --- | --- | --- | --- |
|  | Dialect area | National border | Separation by water | Population difference |
| Log geographic distance | .425 | .350 | .218 | .025 |
| Dialect area |  | .268 | .203 | .022 |
| National border |  |  | .405 | .008 |
| Separation by water |  |  |  | -.008 |

| Supplementary Table 6. Mantel correlations between language-external factors for the *Human body* domain. | | | | |
| --- | --- | --- | --- | --- |
|  | Dialect area | National border | Separation by water | Population difference |
| Log geographic distance | .416 | .309 | .234 | .041 |
| Dialect area |  | .260 | .184 | .038 |
| National border |  |  | .325 | .053 |
| Separation by water |  |  |  | -.005 |

| Supplementary Table 7. Mantel correlations between language-external factors for the *Society and education* domain. | | | | |
| --- | --- | --- | --- | --- |
|  | Dialect area | National border | Separation by water | Population difference |
| Log geographic distance | .421 | .309 | .251 | .051 |
| Dialect area |  | .307 | .244 | .047 |
| National border |  |  | .356 | .046 |
| Separation by water |  |  |  | -.007 |

# Results for partial Mantel correlations across the four domains

We calculated, for each semantic domain separately, partial Mantel correlations between linguistic distance and a number of language-external factors using the *Mantel* function (with 10,000 permutations) in the *ecodist* package (Goslee & Urban 2007) in R. Supplementary Table 8 shows the results for the *Church and religion* domain, Supplementary Table 9 the results for the *Clothing and personal hygiene* domain, Supplementary Table 10 for the *Human body* domain, and Supplementary Table 11 for the *Society and education* domain. These correlational analyses confirmed the findings of the MRM analyses—please refer to the main text for a full discussion.

Supplementary Table 8. Partial Mantel correlations with 95% confidence

intervals and significance levels for the *Church and religion* domain.

|  | *r* | 95% CI | | *p* | |
| --- | --- | --- | --- | --- | --- |
| Log geographic distance | .185 | .165 | .208 | < .001 |  |
| Dialect area | .055 | .032 | .075 | .010 |  |
| National border | .102 | .084 | .121 | < .001 |  |
| Border *distance | -.036 | -.054 | -.018 | .107 |  |
| Separation by water | -.077 | -.097 | -.060 | < .001 |  |
| Water * distance | .091 | .072 | .111 | < .001 |  |
| Population difference | .041 | .010 | .067 | .262 |  |

Supplementary Table 9. Partial Mantel correlations with 95% confidence

intervals and significance levels for the *Clothing and personal hygiene* domain.

|  | *r* | 95% CI | | *p* | |
| --- | --- | --- | --- | --- | --- |
| Log geographic distance | .355 | .336 | .376 | < .001 |  |
| Dialect area | .034 | .018 | .051 | .004 |  |
| National border | .108 | .096 | .121 | < .001 |  |
| Border *distance | -.035 | -.048 | -.021 | .011 |  |
| Separation by water | -.035 | -.047 | -.020 | .003 |  |
| Water * distance | .028 | .014 | .041 | .015 |  |
| Population difference | .000 | -.017 | .022 | .988 |  |

Supplementary Table 10. Partial Mantel correlations with 95% confidence

intervals and significance levels for the *Human body* domain.

|  | *r* | 95% CI | | *p* | |
| --- | --- | --- | --- | --- | --- |
| Log geographic distance | .222 | .206 | .242 | < .001 |  |
| Dialect area | .034 | .018 | .049 | .008 |  |
| National border | .057 | .043 | .073 | .001 |  |
| Border *distance | -.010 | -.026 | .004 | .506 |  |
| Separation by water | -.001 | -.014 | .010 | .961 |  |
| Water * distance | .017 | .006 | .030 | .177 |  |
| Population difference | -.021 | -.039 | -.004 | .416 |  |

Supplementary Table 11. Partial Mantel correlations with 95% confidence

intervals and significance levels for the *Society and education* domain.

|  | *r* | 95% CI | | *p* | |
| --- | --- | --- | --- | --- | --- |
| Log geographic distance | .104 | .091 | .118 | < .001 |  |
| Dialect area | .009 | -.003 | .021 | .398 |  |
| National border | .011 | -.006 | .032 | .372 |  |
| Border *distance | -.007 | -.026 | .011 | .575 |  |
| Separation by water | .014 | -.004 | .029 | .170 |  |
| Water * distance | .001 | -.018 | .018 | .920 |  |
| Population difference | -.018 | -.033 | -.004 | .347 |  |

# Correlations between language-internal factors

| We calculated correlations between the language-internal factors over the four semantic domains combined—see Supplementary Table 12. As the table shows, many of the language-internal factors were highly correlated.  Supplementary Table 12. Correlations between language-internal factors. | | | | | | | | | |
| --- | --- | --- | --- | --- | --- | --- | --- | --- | --- |
|  | Subsections at two levels depth | Subsections at maximum depth | Total number of concepts | Concepts at one level of depth | Concepts at two levels depth | Concepts at maximum depth | Ratio of multi-word concepts | Mean concept length | Median concept length |
| Subsections at one level of depth | .024 | .399 | .685 | .381 | .544 | .327 | .387 | .802 | .845 |
| Subsections at two levels depth |  | .505 | .087 | .062 | .621 | .448 | .001 | .093 | .339 |
| Subsections at maximum depth |  |  | .403 | .436 | .017 | .435 | .181 | .086 | .036 |
| Total number of concepts |  |  |  | .926 | .834 | .647 | .391 | .212 | .314 |
| Concepts at one level of depth |  |  |  |  | .762 | .550 | .703 | .172 | .046 |
| Concepts at two levels depth |  |  |  |  |  | .775 | .312 | .218 | .429 |
| Concepts at maximum depth |  |  |  |  |  |  | .248 | .269 | .307 |
| Ratio of multi-word concepts |  |  |  |  |  |  |  | .802 | .713 |
| Mean concept length |  |  |  |  |  |  |  |  | .947 |
